# Aqueous-Phase Delivery of SLIC-SABRE Hyperpolarized [^15^N_3_]Metronidazole

**DOI:** 10.1021/acs.analchem.6c01373

**Published:** 2026-09-02

**Authors:** Ivan A. Trofimov, Henri de Maissin, Obaid Mohiuddin, Josh P. Peters, Stefan Sell, Anna Samoilenko, Sebastian Lucas, Jochen Leupold, Maxim Zaitsev, Stephan Knecht, Andrey N. Pravdivtsev, Jan-Bernd Hövener, Eduard Y. Chekmenev, Andreas B. Schmidt

**Affiliations:** 1 Division of Medical Physics, Department of Diagnostic and Interventional Radiology, University Medical Center Freiburg, Faculty of Medicine, University of Freiburg, Freiburg 79106, Germany; 2 German Cancer Consortium (DKTK), Partner Site Freiburg, a Partnership between DKFZ and University Medical Center Freiburg, Im Neuenheimer Feld 280, Heidelberg 69120, Germany; 3 Section Biomedical Imaging, Molecular Imaging North Competence Center, Department of Radiology and Neuroradiology, University Hospital Schleswig-Holstein, Kiel University, Kiel 24118, Germany; 4 Department of Chemistry, Integrative Biosciences (Ibio), Karmanos Cancer Institute (KCI), 2954 Wayne State University, Detroit, Michigan 48202, United States; 5 NVision Imaging Technologies GmbH, Ulm 89081, Germany

## Abstract

Metronidazole is an antiprotozoal antibiotic drug holding promise for tumor microenvironment analysis in hyperpolarized MRI due to its hypoxia-sensitive metabolism. Previous studies have explored the ^15^N hyperpolarization of metronidazole using dissolution dynamic nuclear polarization (d-DNP) and signal amplification by reversible exchange (SABRE). Here, we demonstrate that by using spin-lock-induced crossing (SLIC), ^15^N polarization levels up to 10–11% are achieved for 30 mM [^15^N_3_]­metronidazole, improving polarization levels by a factor of 1.4 ± 0.1 compared to polarization levels achieved in conventional SABRE experiments under otherwise the same conditions. This polarization was harnessed to probe the purification of methanolic solutions of hyperpolarized [^15^N_3_]­metronidazole, reducing the methanol concentration by 10-fold in the obtained aqueous solutions of ^15^N-hyperpolarized metronidazole, retaining up to 1/3 of initial polarization. This pilot study marks a substantial step toward the biomedical application of [^15^N_3_]­metronidazole and is promising for the ^15^N-hyperpolarization of other substrates via SLIC and other SABRE strategies.

## Graphical abstract

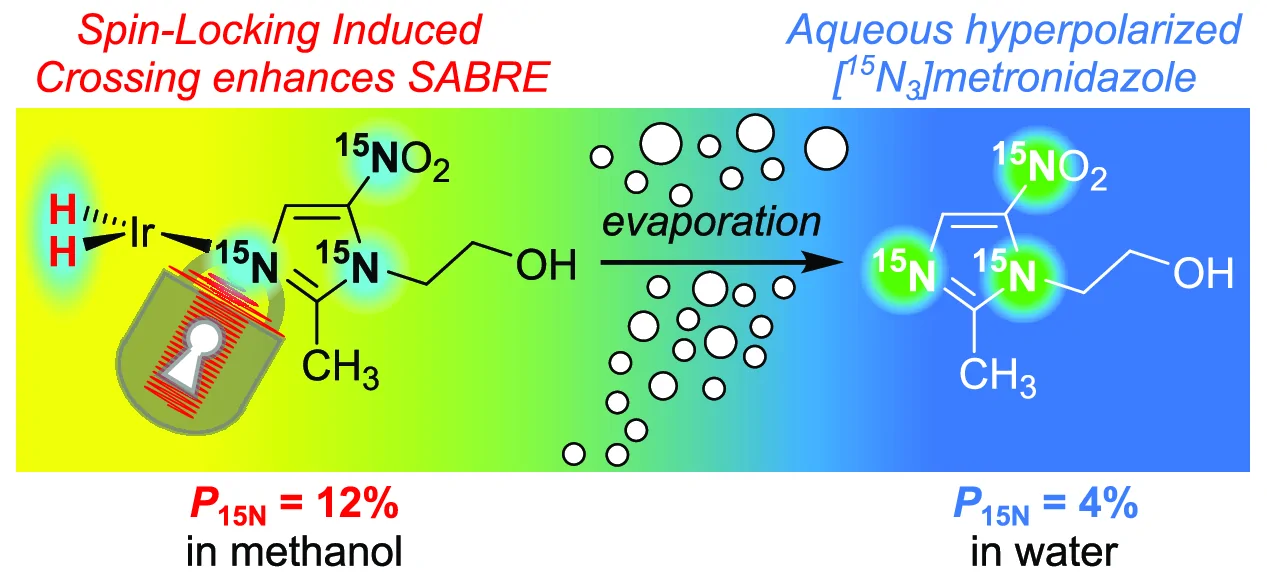

## Introduction

Nuclear magnetic resonance (NMR) spectroscopy and imaging (MRI) of ^13^C and ^15^N nuclei are limited in sensitivity due to their low natural abundance and gyromagnetic ratios relative to ^1^H, despite their fundamental chemical and biomedical significance. Nuclear hyperpolarization, which creates nonequilibrium nuclear spin polarization, has emerged as a powerful tool for significantly increasing the analytical capability of NMR by enhancing signals and reducing measurement times by orders of magnitude.^1^ However, efficiently hyperpolarizing ^15^N nuclei presents a persistent challenge, although it promises value for applications in analytical chemistry, pharmacokinetic studies of drugs, and biomedical imaging. Dissolution dynamic nuclear polarization (d-DNP)^2–6^ is the state-of-the-art method for polarizing ^13^C-labeled metabolites in the liquid state, enabling their use in patient applications.^7,8^ However, reported ^15^N polarizations have been comparatively lower; the highest ^15^N polarizations with d-DNP up to 30% were achieved for [1-^15^N]­nicotinamide after approximately 4 h of microwave irradiation at ∼1.4 K and 6.7 T, followed by dissolution in superheated water.^4^

Parahydrogen-induced polarization (PHIP) uses parahydrogen (the nuclear-spin-singlet state of molecular hydrogen, *p*-H_2_) as a reservoir of latent polarization in chemical reactions.^9–15^ Signal amplification by reversible exchange (SABRE) enables hyperpolarization via reversible binding of *p*-H_2_ and substrate molecules to a transition-metal center.^16–19^ Polarization from *p*-H_2_ is spontaneously transferred to the substrate nuclei, building up in the dissociated, unbound substrate pool when nuclear spin energy-level anticrossing conditions are met.^20^ At submicrotesla magnetic fields, this mechanism induces ^15^N hyperpolarization via the SABRE-SHEATH technique (SABRE in shield enables alignment transfer to heteronuclei).^21,22^ This method was used to produce various HP biomolecules and medicinal drugs,^19,23–25^ which may be useful in MRI and metabolic studies. Recently, SABRE hyperpolarized (HP) [1-^13^C]­pyruvate­(-*d*_3_) has been used in first *in vivo* HP MRI biomedical applications.^26–29^

Metronidazole (MNZ), an antibiotic effective in hypoxic environments, has been a key compound in SABRE-SHEATH studies.^30–33^ Like other nitroimidazole derivatives,^34^ it is of particular interest for sensing oxygen-deficient biological tissues, e.g., for analyzing hypoxic tumor microenvironments. SABRE studies on labeled [^15^N_3_]­metronidazole ([^15^N_3_]­MNZ) showed an efficient network of *J*_15N,15N_-couplings within its SABRE complexes, allowing polarization transfer to all three ^15^N sites (Figure 1a).^31^ Hyperpolarization of [^15^N_3_]­MNZ via SABRE may be carried out in various solvents;^35–37^ for example, recently, studies to assess the applicability of [^15^N_3_]­MNZ for metabolic studies were carried out with production of HP [^15^N_3_]­MNZ in aqueous media using catalyst-separated hyperpolarization through SABRE using a biphasic solvent system CDCl_3_/D_2_O.^36^ An optimized SABRE-SHEATH procedure polarized [^15^N_3_]­MNZ up to *P*_15N_ ≈ 8% on all three nuclei.^37^ Of additional interest, [^15^N_3_]­MNZ has recently been hyperpolarized via d-DNP, achieving up to ∼6%^15^N polarization on each nucleus after dissolution.^5^

**Figure 1:**
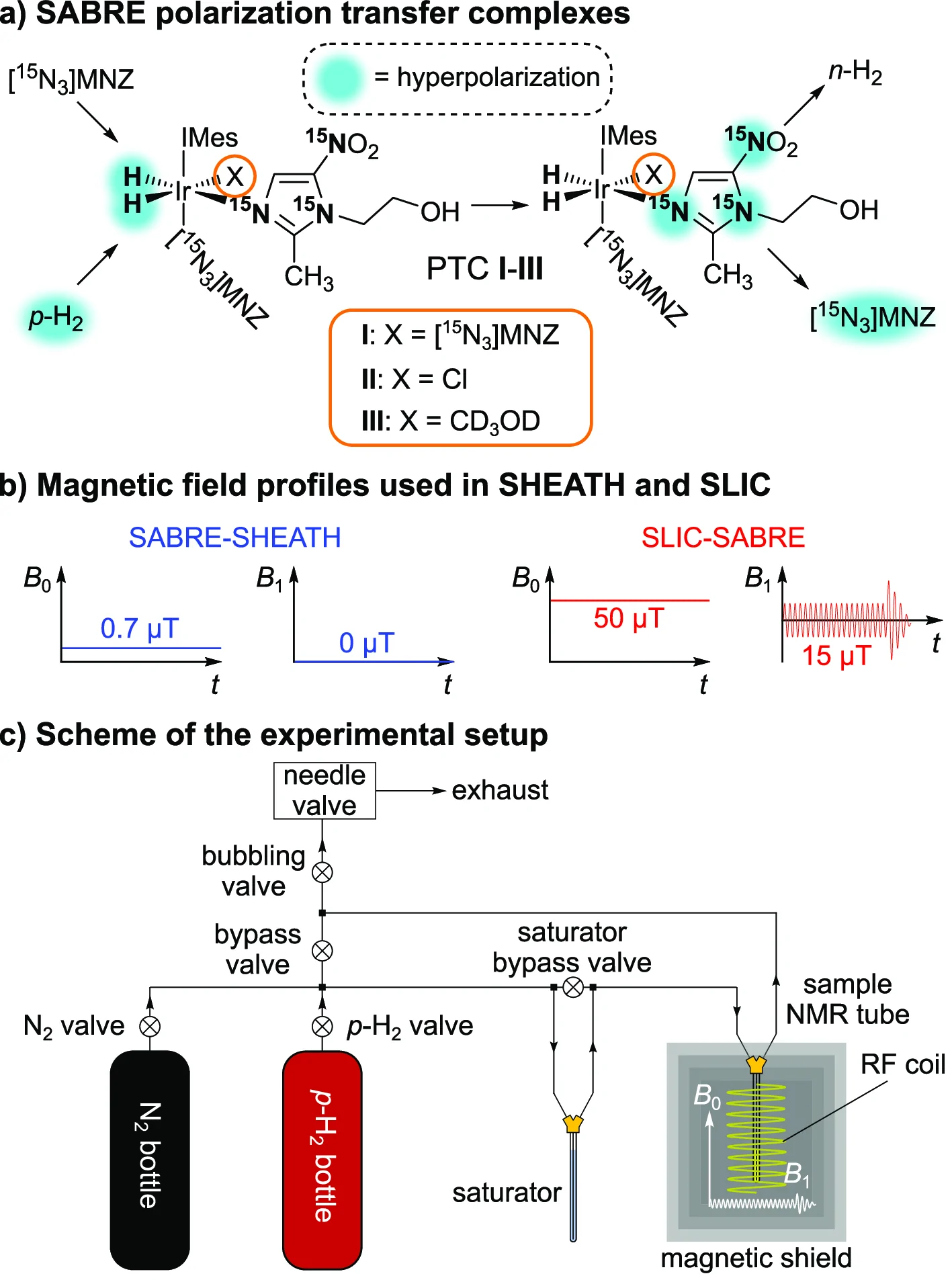
(a) Scheme of SABRE polarization transfer enabled by reversible binding of *p*-H_2_ and [^15^N_3_]­MNZ to the iridium coordination center in polarization transfer complexes (PTCs) **I**–**III** (for assignment see Figure S3 and the accompanying text in the Supporting Information). The orange circle highlights the substituent X that differentiates the PTCs. The teal highlight represents nonthermal polarization transferred via *J*-coupling networks in the PTCs. (b) Linear amplitudes and time dependence of magnetic fields typically applied in the SABRE-SHEATH and SLIC-SABRE protocols. (c) Scheme of the experimental setup used in this work.

Here, we study the application of SLIC-SABRE (spin-lock-induced crossing SABRE)^38–41^ for ^15^N polarization in [^15^N_3_]­MNZ and the subsequent preparation of aqueous ^15^N hyperpolarized solutions via rapid methanol evaporation. SLIC of the energy levels of hydride and the target nuclei is enabled via application of a ^15^N-on resonant radiofrequency (RF) pulse with an amplitude of *B*_1_ in the transverse plane while a longitudinal, static magnetic field (*B*_0_) is maintained; in the end, an adiabatic RF pulse realigns magnetization along the *z*-axis for later NMR signal acquisition (Figure 1b).^42^ Compared to conventional SABRE-SHEATH, this approach provides greater flexibility in the choice of magnetic field during polarization transfer and sample handling. For [^15^N_3_]­MNZ, this flexibility is particularly interesting as relaxation properties are strongly field-dependent and can limit polarization build-up and survival during downstream sample processing.^32^ We therefore employ SLIC-SABRE as a practical route to enhance ^15^N polarization levels in methanol to enable rapid solvent exchange, allowing the delivery of hyperpolarized [^15^N_3_]­MNZ in a physiological aqueous solution with polarization levels suitable for subsequent analytical studies.

## Experimental Section

### Sample Preparation

For preparation of the initial solution, 2.3 mg of SABRE catalyst precursor [IrIMes­(cod)­Cl] (IMes = 1,3-bis­(2,4,6-trimethylphenyl)-1,3-dihydro-2*H*-imidazol-2-ylidene, cod = cycloocta-1,5-diene, synthesized according to a previously published procedure) were dissolved in 900 μL of methanol-*d*_4_ (Sigma-Aldrich, 99% D). Subsequently, 9.6 mg of [^15^N_3_]­metronidazole ([^15^N_3_]­MNZ, synthesized according to the previously published procedure)^43^ was dissolved in 900 μL of degassed methanol-*d*_4_, and the two solutions containing precatalyst and substrate were mixed. The resulting solution contained 2 mM [IrIMes­(cod)­Cl] and 30 mM [^15^N_3_]­MNZ dissolved in methanol-*d*_4_. 600 μL of the prepared solution was then placed in a 5 mm standard high-throughput NMR tube (WG-1000, Wilmad). The NMR tube was connected to the gas lines (N_2_ and H_2_ or *p*-H_2_) and N_2_ gas was bubbled through the solution for several minutes for additional degassing. After degassing, the activation of the solution was initiated by slow bubbling of H_2_ for 2 h at 7.8 bar and room temperature. During the activation (and following experiments), the flow of H_2_ was directed through a saturator NMR tube filled with methanol-*d*_4_, to avoid excessive methanol evaporation from the SABRE solution during bubbling (Figure 1c).^36,37^ After activation, the H_2_ bottle was replaced with a *p*-H_2_ bottle. After these preparations, the setup solution was ready for SABRE experiments.

### SABRE-SHEATH Experiments

An NMR tube containing activated SABRE solution was placed inside a beaker filled with room temperature water (21 °C) held inside a magnetic shield (type MS-1L, Twinleaf LLC, New Jersey, USA) with a static magnetic field *B*_0_ set to 0.71 μT (the measured magnetic field profile is shown in Figure S1a). The field was measured using Fluxgate-Magnetometer Fluxmaster (Stefan-Mayer Instruments, Germany).

Subsequently, bubbling of *p*-H_2_ at 7.8 bar was conducted for 45 s unless stated differently. After the end of the bubbling, the NMR tube was quickly removed from the magnetic shield, dried with a paper towel, and placed in a Bruker Avance Neo 400 WB NMR spectrometer (9.4 T) for subsequent ^15^N signal acquisition and quantification as described in the Supporting Information.

### SLIC-SABRE Experiments

An NMR tube containing an activated SABRE solution was placed in a beaker filled with room-temperature water (21 °C) held inside the static magnetic shield with *B*_0_ field set to 50 μT unless stated otherwise (for the magnetic field profile, see Figure S1b). Subsequently, the bubbling of *p*-H_2_ at 7.8 bar was initiated, and a SLIC radiofrequency pulse (SLIC RF pulse) was applied onto the sample for 77 s (for the parameters of the SLIC RF pulse, see Figure S1b). After 75 s, the bubbling was stopped, and a finishing sweep pulse was applied onto the sample for 2 s to realign the magnetization along *B*_0_. Then, the sample was transferred to the NMR spectrometer in the same way as in SABRE-SHEATH experiments. For optimization of the SLIC-SABRE protocol, certain experimental parameters were varied; for further details, see the Supporting Information.

### NMR Signal Acquisition

Acquisition of the ^15^N NMR signal of the sample was initiated 2–3 s after positioning the NMR tube inside the NMR probe (approximately 10 s after ending the hyperpolarization procedure).

### Purification Protocol

After SLIC-SABRE hyperpolarization, the NMR tube containing the HP sample was disconnected from the setup and heated for 15 s in the field of a ∼100 mT magnet under low vacuum (∼9 mbar; diaphragm pump, 2.4 m^3^/h, MZ2C, Vacuumbrand GmbH, Germany) in hot water (80 or 100 °C). During this exposure to low pressure, 600 μL of D_2_O was gradually added to the solution via pressure imbalance. Between the NMR tube and the diaphragm pump a cold trap cooled with liquid N_2_ was placed. After that, the NMR tube was pressurized back to atmospheric conditions and placed into a holder inside a 7 T Biospec 70/20 MRI scanner (PV6.0.1, Bruker, Germany) for the acquisition of ^15^N NMR spectrum or ^15^N image (TurboRARE pulse sequence); a ^1^H–^15^N quadrature-quadrature coil (V-XQ-HQ-070-02249 V01, Rapid Group GmbH, Germany) was used. ^15^N NMR spectra were acquired for assessment of ^15^N polarization before the image acquisition.

## Results and Discussion

### SLIC-SABRE vs SABRE-SHEATH

For comparison with the current state-of-the-art polarization of [^15^N_3_]­MNZ, we first conducted SABRE-SHEATH experiments using our setup and optimized conditions from the literature. Maintaining 7.8 bar of *p*-H_2_ pressure (∼95% enrichment of *p*-H_2_) and temperature of 21 °C, as optimized in a recent study,^37^ ^15^N polarizations (*P*_15N_) of 5–6% were achieved for the three ^15^N nuclei in [^15^N_3_]­MNZ. The obtained polarization of [^15^N_3_]­MNZ matched that of a 20 mM [^15^N_3_]­MNZ solution in the recent SABRE-SHEATH study.^37^ In agreement with the previous study, *P*_15N_ buildup was rapid, with stationary polarization values reached after about 40 s of *p*-H_2_ bubbling (buildup time *T*_b_ = 18 ± 3 s); under these conditions, *T*_1_ relaxation values were relatively short, ca. 11 s for all three ^15^N nuclei (see Figure S6 in the Supporting Information).

Next, we shifted to SLIC-SABRE. First, the dependence of the ^15^N polarization yield on *B*_0_ and the *B*_1_ was investigated. *P*_15N_ was mostly independent from the *B*_0_ magnetic field in the analyzed range of 35–95 μT, while *B*_1_ was always in resonance with ^15^N (Figure S5). On the other hand, *P*_15N_ appeared sensitive to the *B*_1_ amplitude of the RF pulses, with the optimum found in the range of 12–18 μT, where 10–11% ^15^N polarization was achieved for all ^15^N sites (Figure 2b). The polarization improvement is likely caused by slightly longer relaxation time constants *T*_1ρ_ of 14.9 ± 0.9, 14.4 ± 0.7, and 14.3 ± 0.7 s (^15^N1, ^15^N3 and ^15^NO_2_ sites relaxation in the presence of the SLIC pulse) under SLIC-SABRE conditions compared to the respective *T*_1_ values of 11.7 ± 0.9, 11.2 ± 0.7, and 11.8 ± 0.9 s measured at 0.71 μT under the SABRE-SHEATH conditions (Figure S6). In both SLIC and SHEATH conditions, polarization was distributed so that *P*(^15^N3) < *P*(^15^N1) ≈ *P*(^15^NO_2_). The ^15^N3 site likely has the least polarization because it relaxes faster due to stronger interactions with labile H/D atoms of the solvent^44^ and quadrupolar ^191^Ir and ^193^Ir nuclei^45^ in the PTCs **I**–**III**. The *T*_1_ relaxation times of all ^15^N sites at 50 μT in absence of the SLIC pulse are dramatically longer (120 ± 8, 121 ± 9, and 121 ± 8 s for ^15^N1, ^15^N3, and ^15^NO_2_ sites, respectively; Figures 2d and S6), in general agreement with previous *T*_1_-data for ^15^N_2_,^13^C_2_-labeled MNZ. The dramatic rise of the *T*_1_ relaxation times in static magnetic field may be attributed to suppression of interactions of ^15^N nuclei with the quadrupolar Ir nuclei at 50 μT. However, large discrepancy between *T*_1_ and *T*_1ρ_ relaxation times should also be noted; previously, *T*_1_ > *T*_1ρ_ relationship was reported for ^15^N nuclei in pyridine (7.8 s > 5 s)^41^ and ^13^C nuclei in [1-^13^C]­pyruvate (159 s > 113 s).^26^ Here *T*_1ρ_ is dramatically shorter than *T*_1_. To the best of our knowledge, this study is a first report on SLIC-SABRE hyperpolarization of a substrate with multiple heteronuclear labels of the same kind (in this case, several ^15^N labels), so, we tentatively suggest that either chemical inequivalence between the ^15^N nuclei (Δδ ≈ 100 ppm) or homonuclear ^2^ *J*_15N,15N_ couplings (≈ 1.5 Hz) may introduce difference in evolution of inequivalent nuclei during SLIC pulse application that enhance *T*_1ρ_ relaxation. Investigation of the reason for such strong effect of the SLIC pulse on *T*_1ρ_ in this SABRE system is the direction of our future work.

**Figure 2:**
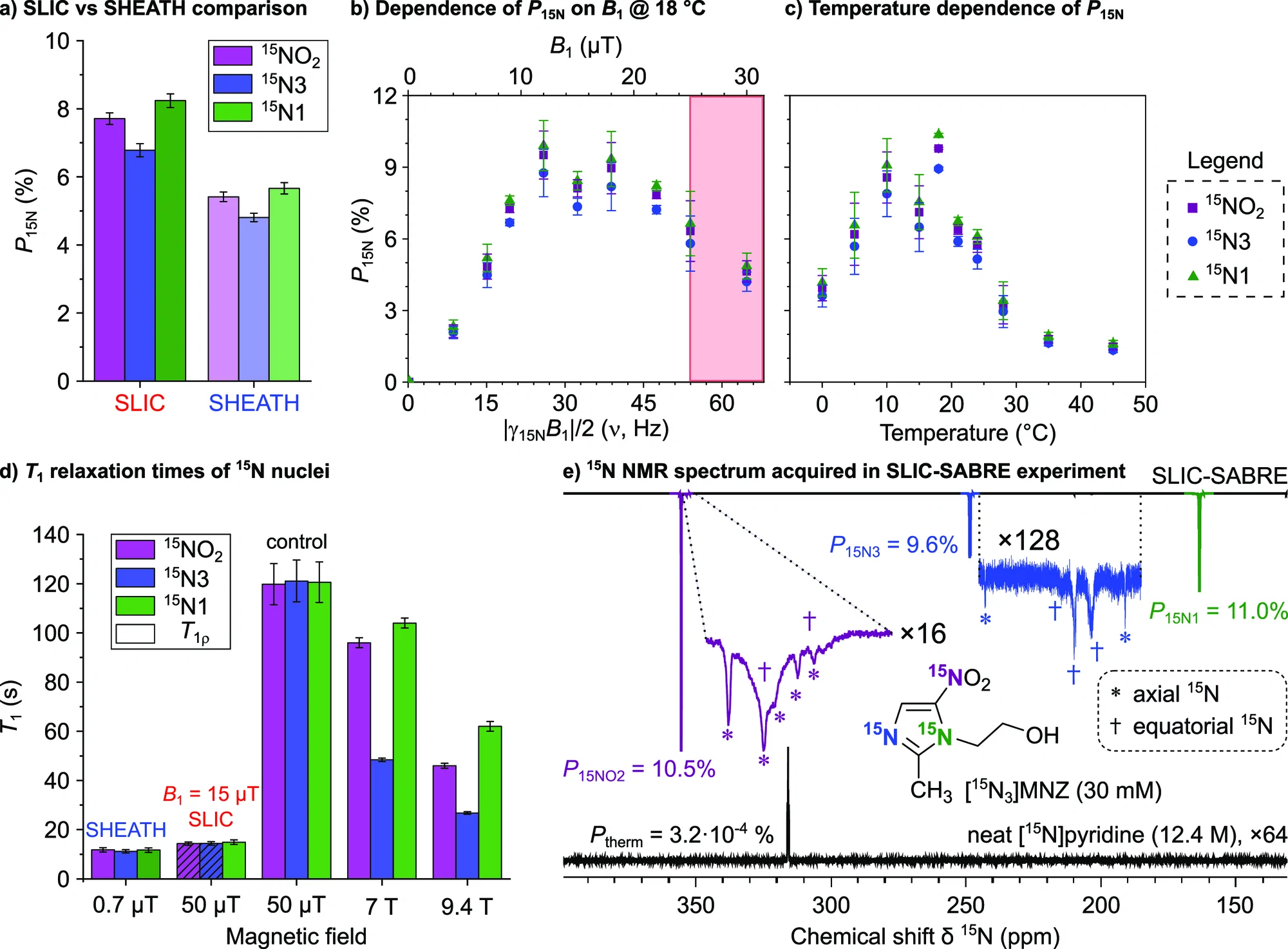
(a) Comparison of average *P*_15N_ achieved after buildup in SABRE-SHEATH (*B*_0_ = 0.71 μT) and SLIC-SABRE conditions (*B*_0_ = 50.1 μT, *B*_1_ = 12 μT) obtained by fitting experimental buildup data (see Figure S4). (b) Dependence of *P*_15N_ achieved in SLIC-SABRE conditions utilizing different *B*_1_ fields (maintaining *B*_0_ = 50.1 μT). The deviations were estimated by repeating each experiment 3 times. The region highlighted with red denotes the range of *B*_1_ where the final SLIC RF pulse was slightly distorted due to power limitations of the *B*_1_ channel. The lower *y*-axis is recalculated from *B*_1_ using reduced γ_15N_ = 4.316 Hz/μT. (c) Dependence of *P*_15N_ achieved in SLIC-SABRE conditions at different temperatures (*B*_0_ = 50.1 μT, *B*_1_ = 12 μT). The deviations were estimated by repeating each experiment 3 times. (d) Bar plot with *T*_1_ relaxation times of the ^15^N nuclei in [^15^N_3_]­MNZ in the SABRE solution. The deviations were estimated by fitting of data points by exponential function (see Figures S5 and S6). (e) Typical ^15^N NMR spectrum of [^15^N_3_]­MNZ hyperpolarized via SLIC-SABRE (*B*_0_ = 50.1 μT, *B*_1_ = 12 μT) and thermally polarized neat [^15^N]­pyridine (black). The insets show NMR signals from the [^15^N_3_]­MNZ molecules that are bound in the PTCs **I**–**III**.

Next, we investigated the dependence of SLIC-SABRE efficiency on the temperature, which directly affects the exchange rates between bound and free H_2_ and [^15^N_3_]­MNZ molecules. The polarization reached a maximum at approximately 10–18 °C (Figure 2c), indicating the same temperature dependence previously reported for SABRE-SHEATH.^37^

The ^15^N NMR spectra of [^15^N_3_]­MNZ obtained under SLIC-SABRE conditions show contributions from multiple PTCs, some strongly broadened and partially overlapping with other signals (Figure 2e). Along with the species **I**–**III** (identified in previous works^36,37,46^ ), at least one more complex seems to be responsible for polarization transfer (as indicated by the amount of different ^15^NO_2_ and ^15^N3 signals corresponding to the bound species); however, ^1^H NMR analysis shows that its concentration is fairly small compared to **I**–**III** (Figures S2 and S3). The dominant signals from the free substrate could be reliably quantified and showed that SLIC-SABRE reproducibly enhanced the polarization of all three ^15^N sites by a factor of 1.4 ± 0.1 (Figure S4) compared to SABRE-SHEATH–^15^N polarization levels up to 10–11% were achieved. The driver for this increase of *P*_15N_ levels achieved in SLIC-SABRE experiments compared to SABRE-SHEATH is longer relaxation time of ^15^N nuclei in the rotating frame during SLIC pulse application (*T*_1ρ_ ≈ 14–15 s) compared to spin–lattice relaxation time in SHEATH conditions (*T*_1_ ≈ 11–12 s). This increase in *T*_1ρ_ is a net result of increased static *B*_0_ field and application of SLIC pulse.

### Probing Purification of SABRE Solutions

For biomedical applications of SABRE-hyperpolarized compounds, transfer to an aqueous phase with methanol and the iridium-based hyperpolarization catalyst reduced below toxic levels is needed. This purification process requires time during which hyperpolarization is lost to relaxation, and a major challenge is to sustain a maximum fraction of precious transient hyperpolarization. Strategies that have been investigated for MNZ purification include catalyst scavenging to reduce iridium levels^47^ and phase separation using biphasic systems like CDCl_3_/D_2_O, which establish purification by accumulating hyperpolarized [^15^N_3_]­MNZ in the aqueous phase.^36,48^ Rapid methanol evaporation was recently shown to rapidly reduce methanol and catalyst levels in the aqueous phase by over 100 times but has been studied only for the purification of [1-^13^C]­pyruvate-*d*_3_.^26^ This approach has enabled retention of 1/3 of the initial HP ^13^C NMR signal and has enabled first *in vivo* studies using SABRE [1-^13^C]­pyruvate-*d*_3_ in both healthy mice and a transgenic mouse model of breast cancer.^26,29^

Polarization losses due to *T*_1_ relaxation are reduced when conducting the sample workup at fields with long *T*_1_ relaxation times. In earlier works,^35,49^ it was shown that *T*_1_ of the ^15^N nuclei in nonlabeled and ^15^N_2_-labeled MNZ is longest at 0.1–1 T. However, in these works, relaxation of MNZ was studied in SABRE solutions, i.e., in the presence of the Ir catalysts. While purification starts from a solution containing Ir catalysts, it is important to investigate relaxation of MNZ in their absence since in purified state the catalyst should be removed. Additionally, the relaxation dynamics of [^15^N_3_]­MNZ in wide range of magnetic fields was not studied previously. To bridge this gap and better assess the range of magnetic fields in which purification should be conducted, we measured nuclear magnetic relaxation dispersion (NMRD) of [^15^N_3_]­MNZ in methanol-*d*_4_ and water-*d*_2_ in magnetic fields of 7.8 μT to 9.4 T using a mechanical magnetic field cycling (MFC) device.^50^

In both methanolic and aqueous solutions we have prepared saturated solutions of [^15^N_3_]­MNZ for the NMRD studies: 39 mM in water-*d*_2_ and 107 mM in methanol-*d*_4_. The concentrations of [^15^N_3_]­MNZ were measured by comparing signals in the ^15^N NMR spectra of the prepared samples to the reference signal of 101 mM [^15^N]­pyridine in water (see the Supporting Information for more details). In aqueous systems, we have probed the solutions without additives (Figure 3a) and with added Tris buffer (pH 7.5, Figure 3b). In low fields (<1 mT), all ^15^N sites experienced quite fast *T*_1_ relaxation (^15^NO_2_, ∼10 s; ^15^N3, ∼2 s; ^15^N1, ∼5 s). *T*_1_-relaxation in these fields was significantly prolonged in the presence of Tris buffer (^15^NO_2_, ∼40 s; ^15^N3, ∼10 s; ^15^N1, ∼20 s), quite likely as a result of chemically induced deceleration of nuclear spin relaxation (CIDER) effect.^44^ The concentration of [^15^N_3_]­MNZ in the buffered solution was determined to be 38.5 mM, slightly lower than in the water-*d*_2_ solution without the buffer. The *T*_1_ relaxation times of both ^15^N3 and ^15^N1 reached maxima of 100–200 s in magnetic fields of 0.3–2 T in both buffered and nonbuffered solutions. Further decrease of *T*_1_ of all ^15^N sites in high magnetic fields (>2 T) is explained by the field dependent contribution of the chemical shift anisotropy, as it is proportional to the square of the magnetic field.^45^

**Figure 3:**
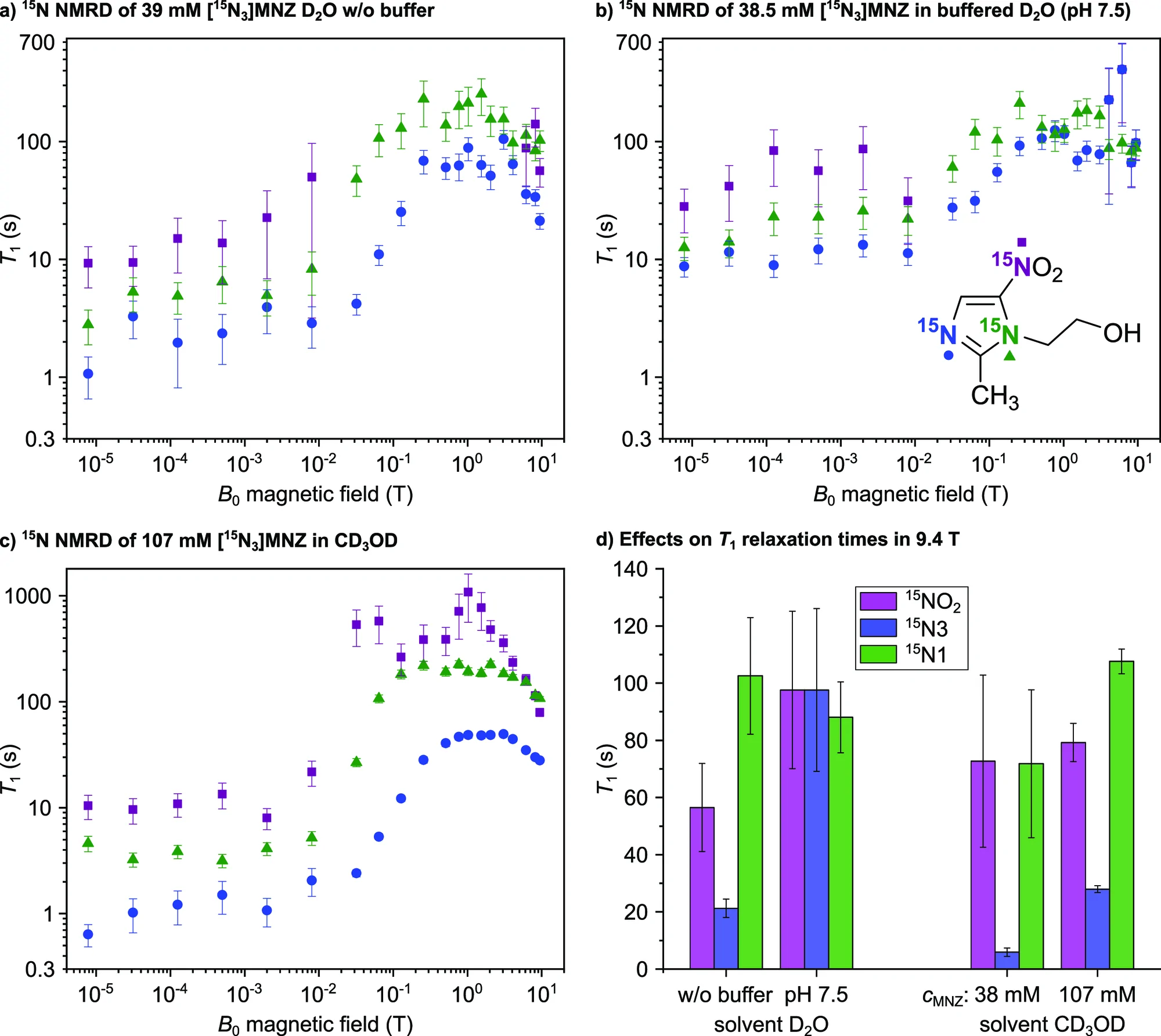
^15^N NMRD for [^15^N_3_]­MNZ. Field dependence of longitudinal relaxation time constant *T*_1_ was investigated in two different solvents, namely, (a) water-*d*_2_ solution (*c*_MNZ_ ≈ 39 mM), (b) buffered water-*d*_2_ solution (*c*_MNZ_ ≈ 38.5 mM, 62.1 mM Tris, and 1.4 mM EDTA), and (c) methanol-*d*_4_ solution (*c*_MNZ_ ≈ 107 mM). For measurements of ^15^N3 and ^15^N1 in water-*d*_2_ and for all nuclei in methanol-*d*_4_, the signals were enhanced using the SWAP pulse sequence.^51^ Note that *T*_1_ of the ^15^NO_2_ group in the water-*d*_2_ at *B*_0_ > 10^–2^ T was not determined because of low SNR in the experiment. The same data are presented in the Supporting Information in numerical form (Table S2). (d) Comparison of *T*_1_ relaxation times in magnetic field of 9.4 T to showcase CIDER and solvent effects.

In methanol-*d*_4_, we observed *T*_1_ values for the ^15^N1 site between 181 and 225 s, whereas ^15^NO_2_ reaches up to 400–1100 s in methanol-*d*_4_ in magnetic fields of 0.1–2.0 T (Figure 3c). The ^15^N3 nucleus, however, exhibits a relatively short *T*_1_ of ∼40 s in methanol-*d*_4_. In lower magnetic fields of 10^–5^–10^–3^ T, the *T*_1_ relaxation times of all nuclei decrease significantly, reaching ∼10 s for the ^15^NO_2_, ∼3 s for the ^15^N1, and ∼1 s for the ^15^N3 sites. The shorter *T*_1_ of the ^15^N3 site compared to those of ^15^N1 and ^15^NO_2_ is likely due to the interactions of the lone electronic pair of this nucleus with the labile deuterons of methanol-*d*_4_.^4,44^ The decrease of *T*_1_ of all ^15^N sites in high magnetic fields (>2 T) is again explained by the field dependent contribution of the chemical shift anisotropy. Thus, these results are in general agreement with earlier data reported by Kiryutin et al. in SABRE solution.^35^

As we examined *T*_1_-relaxation of [^15^N_3_]­MNZ in methanol-*d*_4_ at higher concentration than in water-*d*_2_, differences in *T*_1_ values between these samples may not be attributed to explicitly to either solvent or concentration effects. To distinguish between these effects on the *T*_1_ relaxation of [^15^N_3_]­MNZ, we have also attempted to acquire NMRD curve for 38 mM solution of [^15^N_3_]­MNZ in methanol-*d*_4_; however, we were unsuccessful and did not manage to detect signals after relaxation in any field other than 9.4 T (Figure S9) due to low signal-to-noise ratio (SNR). Nonetheless, comparison of the relaxation rates in this high field (Figure 3d) demonstrates that significant concentration effect on *T*_1_ relaxation times of [^15^N_3_]­MNZ is present (^15^N3, 5.9 ± 1.5 s vs 27.9 ± 1.2 s, and ^15^N1, 72 ± 26 s vs 108 ± 4 s at 38 and 107 mM, respectively). This concentration effect is likely also caused by CIDER effect, as similar *T*_1_ increase was reported for concentrated solutions of [1-^15^N]­nicotinamide, where the same molecules essentially shield each other from the interactions detrimental to *T*_1_.^44^

In terms of field dependence, the trends were the same as in methanol-*d*_4_: the longest *T*_1_ of ^15^N1 and ^15^N3 nuclei in water was also observed in the range of magnetic fields of 0.1–2 T. It means that ideal magnetic fields for the sample workup should be between 0.5 and 2 T; currently we used lower 0.1 T fields, which corresponds to rather short *T*_1_ in methanol-*d*_4_ but prolonged *T*_1_ in buffered aqueous solution.

Hence, to study methanol evaporation for the purification of ^15^N-hyperpolarized solutions of [^15^N_3_]­MNZ, we implemented this method at a magnetic field of ∼100 mT (Figure 4a). Methanol evaporation (15 s) was investigated at two different temperatures, 80 and 100 °C, under a low vacuum (9 mbar). When heated to 100 °C, methanol was evaporated down to 0.05 ± 0.02 mol % or 32 ± 12 mM in 15 s (see the Supporting Information for calculations of methanol-*d*_4_ content), while the iridium catalyst precipitated. However, the initial ^15^N polarization was largely lost, decaying to ca. 0.05% or lower, with the remaining signal sometimes below the detection limit. In contrast, when the purification was performed at 80 °C, only minor precipitation was observed (also supported by the detection of ^15^N NMR signals corresponding to the Ir-bound [^15^N_3_]­MNZ molecules and the solution remaining pronouncedly yellow). The remaining methanol-*d*_4_ content was significantly higher, 6.0 ± 0.4 mol % or 3.2 ± 0.2 M. Interestingly, a significantly higher fraction of the initial ^15^N-polarization was retained (Figure 4b): a retention factor of over 1/3 was observed for all ^15^N1, ^15^N3, and ^15^NO_2_ sites. Hence, we hypothesize that formation of the precipitate may be detrimental to retention ^15^N polarization, potentially due to relaxation in the precipitate-bound solid state.^52^ In all cases, the [^15^N_3_]­MNZ concentrations in the final solution were reduced compared to the starting concentration due to sample loss and splashing; while at 80 °C the splashing was moderate and 20 ± 3 mM of [^15^N_3_]­MNZ was preserved, the effect was stronger at 100 °C and led to modest 7.1 ± 0.3 mM of residual [^15^N_3_]­MNZ. Probably due to the same reason, larger losses of the solution were observed at 100 °C (≈250 μL of residual solution) than at 80 °C (≈450 μL). The results of this study are summarized in Table 1.

**Table 1:** Residual Methanol-*d*_4_ concentration *c*(CD_3_OD) and Polarization Retention Factors of the ^15^N Sites Achieved by Methanol-*d*_4_ Evaporation for 15 s at 80 and 100 °C

	evaporation temperature, °C	
	80	100
residual *c*(CD_3_OD), mol/L	3.2 ± 0.2	0.032 ± 0.012
residual *c*([^15^N_3_]MNZ), mmol/L	20 ± 2	7.1 ± 0.3
final sample volume, μL	≈450 ± 40	≈250 ± 75
*P*_15N_ retention factors		
^15^NO_2_	0.40 ± 0.10	<0.01
^15^N3	0.37 ± 0.08	<0.01
^15^N1	0.39 ± 0.09	<0.01

**Figure 4:**
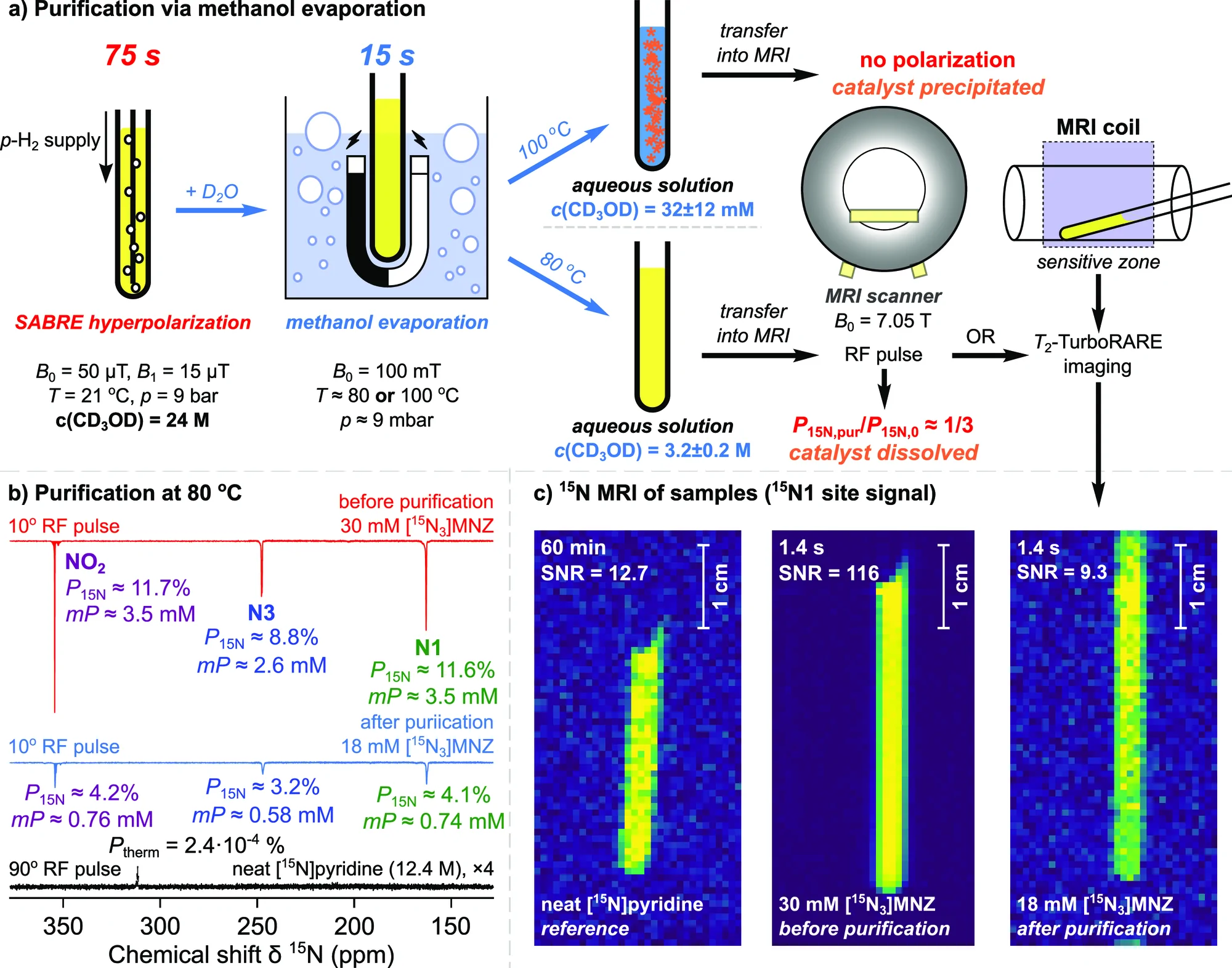
(a) Scheme of purification experiments and corresponding chemical changes in the solution. (b) Typical ^15^N NMR spectra (obtained on the 7.05 T MRI scanner) of [^15^N_3_]­MNZ hyperpolarized via SLIC-SABRE (*B*_0_ = 50.1 μT, *B*_1_ = 15 μT, red) and after injection of D_2_O and 15 s evaporation of methanol-*d*_4_ at 80 °C (light-blue). In addition to *P*_15N_, molar polarization *mP* (product of concentration and polarization) is stated to better reflect simultaneous signal and concentration losses. (c) ^15^N MRI images of hyperpolarized 30 mM [^15^N_3_]­MNZ in SABRE solution (single scan, 1.4 s), hyperpolarized 18 mM [^15^N_3_]­MNZ in aqueous solution after purification at 80 °C (single scan), and (c) neat (12.4 M) [^15^N]­pyridine (128 averages, 60 min). TurboRARE pulse sequence (FOV, 5 × 2.5 cm^2^; matrix, 64 × 32; pixel size, 0.8 × 0.8 mm^2^; RARE factor 64; TR = 330 ms; TE = 122 ms) was used for image acquisition.

The ^15^N polarization of [^15^N_3_]­MNZ in the aqueous solution after purification at 80 °C appeared sufficient for ^15^N MRI: a single MRI scan of a hyperpolarized sample after methanol evaporation provided SNR of 9.3 comparable to that in the image of neat (12.4 M) [^15^N]­pyridine used as a thermal reference acquired over 1 h (128 averages, Figure 4c). TurboRARE pulse sequence was used for image acquisition, harnessing the hyperpolarized signal of ^15^N1 nuclei using spectrally selective excitation pulses.

### Outlook

These results are already promising, however, the long activation times (2 hours) needed to prepare SABRE HP [^15^N_3_]­MNZ are challenging the optimization. While in future implementations this may be circumvented by activating larger MNZ batches, we discuss several possible strategies for improving the methanol evaporation implementation for HP [^15^N_3_]­MNZ in the following:

First, on the basis of our observations, we suggest a hypothesis that the formation of catalyst precipitate during the purification of [^15^N_3_]­MNZ is detrimental to ^15^N polarization levels. To avoid precipitate formation, methanol evaporation may be combined with subsequent catalyst scavenging using functionalized resins,^47,53^ leading to a catalyst-free aqueous solution of HP [^15^N_3_]­MNZ.

Second, the amount of residual ^15^N polarization may be improved by injecting various additives such as (i) ascorbate (used as an oxygen scavenger in PHIP experiments),^54^ (ii) agents for chemically induced deceleration of relaxation like urea (prevents loss of polarization via interactions of lone pair of the ^15^N3 atom with labile protons/deuterons in the solvent),^44^ (iii) pH buffers like Tris or PBS,^44^ or (iv) quenchers of chemical exchange on SABRE catalyst like 2,2′-bipyridine^55^ before evaporation.

Third, the issue of high residual alcohol content may be mitigated by optimizing the evaporation duration and temperature, or by using ethanol rather than methanol as the solvent, thereby allowing higher residual alcohol content in the solution after purification, because ethanol is less toxic. It was recently shown that efficient SABRE-hyperpolarization of [^15^N_3_]­nimorazole, a [^15^N_3_]­MNZ analog, is possible in nondeuterated ethanol,^37^ hence, usage of ethanol as a starting solvent may indeed be promising for some of the nitroimidazoles.

Finally, the NMRD demonstrated the optimal range for polarization retention, 0.5–2 T. The impact of the previously discussed manipulations and samples following each workup should also be studied in the context of finding the optimal field for *T*_1_. More powerful magnets would be necessary to deliver magnetic fields more suitable for polarization retention than those currently used.

SLIC-SABRE approach was earlier shown to improve the polarization levels of clinically relevant ^15^N compounds, including other nitroimidazole derivatives.^41^ Its combination with methanol evaporation presented in this work, other purification approaches discussed above, and ^15^N-labeling paves the way toward biocompatible formulations of ^15^N-hyperpolarized agents. Looking ahead, the SLIC-SABRE approach may be further extended to other ^15^N-compounds, including other nitroimidazole derivatives, ^15^N hyperpolarization of which was previously explored using SABRE-SHEATH.^56–60^ Additionally, deuteration of the methyl group in the ^15^N_3_-labeled probe may increase polarization levels achieved in SLIC-SABRE experiments further similar to the studies using sodium pyruvate-*d*_3_.^40^ Further improvements on the SLIC strategy are also possible, as evident from large difference between *T*_1_ and *T*_1ρ_ for [^15^N_3_]­MNZ reported here and results from recent literature.^61^ Nitroimidazoles are interesting probes due to their potential for *in vivo* imaging and pharmacokinetic studies. Active uptake of MNZ in hypoxic tissues makes it particularly promising for visualizing hypoxic regions.^62^ Notably, structurally similar ^18^F-fluoromisonidazole is used in positron emission tomography for visualizing hypoxic tissue.^34^ Hypoxia plays a crucial role in conditions such as cancer, where tumor hypoxia can contribute to radiation resistance.

## Conclusions

In summary, we have demonstrated significantly improved ^15^N hyperpolarization of [^15^N_3_]­MNZ using SLIC-SABRE compared to SABRE-SHEATH (improvement factor of 1.4 ± 0.1). Furthermore, purification of methanolic solution of hyperpolarized [^15^N_3_]­MNZ via mixing with water and evaporation of methanol under vacuum was probed for the first time. Purification at 100 °C removed almost all methanol, but polarization decayed potentially because of enhanced relaxation. Purification at 80 °C, on the other hand, showed good polarization retention (>1/3) but residual methanol content remained high (over 1 M), as well as content of Ir-species (as evident from lack of precipitation during methanol removal), exceeding biocompatible levels and encouraging test of modifications to the current purification protocol. Nevertheless, this advancement brings SABRE of [^15^N_3_]­MNZ closer to biomedical application and may be expanded to other ^15^N labeled substrates, offering an affordable analytical tool for investigating pharmacokinetics, probing enzymatic reactions, and potentially biomedical imaging of tumor hypoxia.

## Supplementary Material



## Notes

Expanded materials and methods, experimental results, and data processing (PDF)

## Abbreviations

cod: cycloocta-1,5-diene
d-DNP: dissolution dynamic nuclear polarization
EDTA: ethylenediamine­tetraacetic acid
HP: hyperpolarized
IMes: 1,3-bis­(2,4,6-trimethylphenyl)-1,3-dihydro-2*H*-imidazol-2-ylidene
MFC: magnetic field cycling
MNZ: metronidazole
MRI: magnetic resonance imaging
NMR: nuclear magnetic resonance
NMRD: nuclear magnetic relaxation dispersion
PHIP: parahydrogen-induced polarization
PTC: polarization transfer complex
RF: radiofrequency
SABRE: signal amplification by reversible exchange
SABRE-SHEATH: SABRE in (magnetic) shield enables alignment transfer to heteronuclei
SLIC: spin-lock-induced crossing
SNR: signal-to-noise ratio
