# Supplementary material for: Aqueous-Phase Delivery of SLIC-SABRE Hyperpolarized [15N3]Metronidazole
Source: Anal Chem. 2026 Sep 2;98(39):28656–65. doi: 10.1021/acs.analchem.6c01373 (PMC13647987; doi:10.1021/acs.analchem.6c01373)
Supplement: Supplementary file 1 [file ac6c01373_si_001.pdf]

Supporting Information for

Aqueous-Phase Delivery of SLIC-SABRE Hyperpolarized [$^{15}N_3$]Metronidazole

*Ivan A. Trofimov[1], Henri de Maissin[1,2], Obaid Mohiuddin[1], Josh P. Peters[3], Stefan Sell[1], Anna Samoilenko[4], Sebastian Lucas[5], Jochen Leupold[1], Maxim Zaitsev[1], Stephan Knecht[5], Andrey N. Pravdivtsev[3], Jan-Bernd Hövener[3], Eduard Y. Chekmenev[4], Andreas B. Schmidt[1,2,3]**

[1] Division of Medical Physics, Department of Diagnostic and Interventional Radiology, University Medical Center Freiburg, Faculty of Medicine, University of Freiburg, Freiburg 79106, Germany

[2] German Cancer Consortium (DKTK), partner site Freiburg, a partnership between DKFZ and University Medical Center Freiburg, Im Neuenheimer Feld 280, Heidelberg 69120, Germany

[3] Section Biomedical Imaging, Molecular Imaging North Competence Center, Department of Radiology and Neuroradiology, University Hospital Schleswig-Holstein, Kiel University, 24118 Kiel, Germany

[4] Department of Chemistry, Integrative Biosciences (Ibio), Karmanos Cancer Institute (KCI), Wayne State University, Detroit, Michigan 48202, United States

[5] NVision Imaging Technologies GmbH, 89081 Ulm, Germany

Table of contents

Materials and methods 2
Data processing 3
Hydride complexes in activated SABRE solution of [$^{15}N_3$]MNZ 4
^{15}N polarization levels dependence on magnetic field B_0 in SLIC-SABRE 6
T_1 relaxation times of ^{15}N nuclei in [$^{15}N_3$]metronidazole 7
^{15}N NMRD measurements 7
Quantification of methanol content after purification 9
Parahydrogen enrichment quantification 11
References 12

Materials and methods

List of parameter variations in SABRE experiments

- Polarization buildup experiments: Using SABRE-SHEATH and SLIC-SABRE hyperpolarization protocols, the duration of the bubbling of p-H_2, and B_1 application in case of SLIC-SABRE, was varied.

- Polarization relaxation studies: To analyze T_1 or $T_{1\rho}$ relaxation at the B_0 fields applied during SABRE-SHEATH or SLIC-SABRE hyperpolarization, a variable delay was added between the end of the p-H_2 bubbling and the removal of the NMR tube from the magnetic shield. Note that for $T_{1\rho}$ measurements, the SLIC RF pulse was turned on during this delay and the adiabatic pulse aligning the magnetization along B_0 was applied right before sample transfer to the NMR system. For relaxation study at 9.4 T, 9° RF pulses (acquisition time of 3 s) were applied to the sample with 15 s repetition time.

- Polarization dependence on B_0: SLIC-SABRE efficiency was investigated at different longitudinal magnetic fields B_0; for each field, the frequency of the SLIC RF pulse was adjusted to the Larmor frequency of the ^{15}N nuclei at the corresponding field.

- Polarization dependence on B_1: SLIC-SABRE investigated for different amplitudes of the SLIC RF pulse B_1 at fixed B_0 of 50.1 µT.

- Temperature dependence of SLIC-SABRE: The temperature was varied and held stable during the experiments by mixing appropriate amounts of room temperature water and ice inside the beaker.

Magnetic field profiles in the magnetic shield

To maintain a stable longitudinal magnetic field B_0 and to generate SLIC RF pulses with amplitude B_1 the Twinleaf shield was used. For maintenance of B_0 magnetic field the HMP2030 Programmable Power Supply unit (Rohde & Schwarz GmbH, Germany) was used. SLIC RF pulses were generated using a multifunction I/O device (USB-6363, National Instruments, USA); the SLIC RF pulses were amplified by using 12 W amplifier (Kemo-Electronic GmbH, Germany). In SABRE-SHEATH experiments, the magnetic field profile created is shown in Figure S1a. Notably, it showed no zero-crossing along the z-axis which was observed in some implementations due to multilayer µ-metal shielding of the setup from the Earth's magnetic field. In SLIC-SABRE experiments, a B_0 field drop down to ~10 µT was observed along the z-axis attributable to the µ-metal shielding (Figure S1b). As the range of homogeneous B_0 magnetic field (total deviation <1%) along the z-axis was small using the MS-1L magnetic shield, the flow rate of p-H_2 in SABRE experiments was limited (see schematic in Figure S1a).

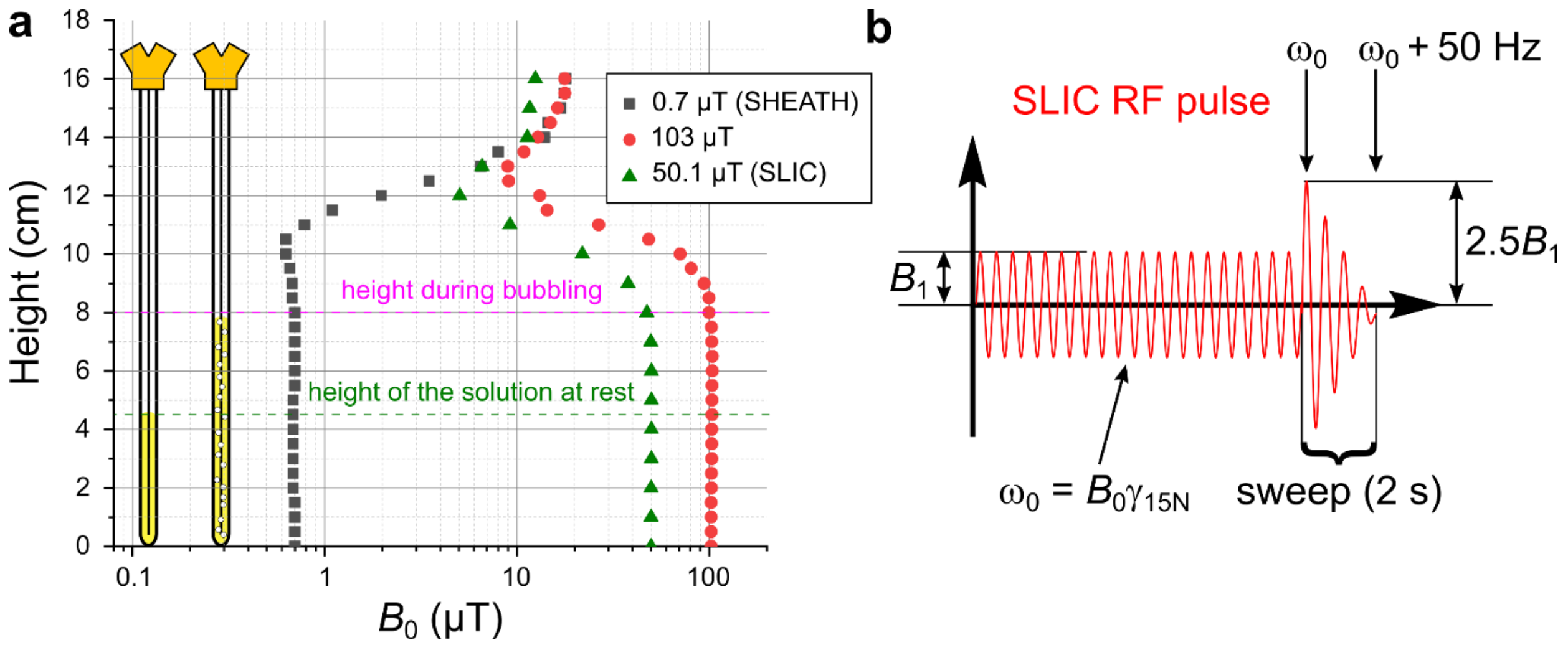

Figure S1. (**a**) B_0 magnetic field profile present in SABRE-SHEATH (black squares; 0.70 µT, field homogeneity over sample height of ±0.01 µT, i.e. ~1%) and SLIC-SABRE experiments (green triangles; 50.1 µT, field homogeneity over sample height of ±0.5 µT, i.e. ~1%). Similar B_0 magnetic field profile is demonstrated for 103 µT (red circles) to showcase that similar magnetic field homogeneity is observed even in higher fields. For reference, schematic NMR tubes are presented with marks for the height of the solution at rest (green line, ~4.5 cm) and during bubbling of p-H_2 (pink line, 7-8 cm). (**b**) Scheme of the SLIC RF pulse with the adiabatic pulse played out at the end to obtain longitudinal ^{13}C magnetization and used parameters (buildup and sweep amplitude and their relation, pulse frequency and sweep duration).

Data processing

^{15}N signal enhancement: The ^{15}N NMR spectra obtained at 9.4 T (400 MHz NMR spectrometer) were processed the following way. Integral intensities I_{HP} of the NMR signals of ^{15}N nuclei of [$^{15}N_3$]MNZ were at first recalculated to ^{15}N NMR signal enhancement factors $SE_{15\mathrm{N}}$ as

$$SE_{15\mathrm{N}} = \frac{I_{\mathrm{HP}}}{I_{\mathrm{ref}}} \cdot \frac{c_{\mathrm{ref}}}{c_{\mathrm{MNZ},i}} \cdot \frac{h}{h_0} \cdot f$$

where I_{ref} is the integral of ^{15}N NMR signal of the reference sample (neat [^{15}N]pyridine) thermally equilibrated at the 9.4T detection field, c_{ref} is the concentration of the reference nuclei (12.4 M), $c_{\mathrm{MNZ},i}$ is the concentration of equatorially or axially bound or non-bound [$^{15}N_3$]MNZ in the solution (see the following paragraph for calculation of $c_{\mathrm{MNZ},i}$), h is the height of the SABRE solution inside the NMR tube, h_0 is the initial height of the SABRE solution inside the NMR tube (with their ratio accounting for changes in concentration due to evaporation), and f is the spatial factor needed to account for presence of the catheter in the SABRE solution (and lack thereof in the reference sample). The spatial factor f is determined by the inner diameter of the NMR tube (ID_{tube}, 4.0 mm), outer diameter of the catheter (OD_{cat}, 0.5 mm ID 1/16" PTFE, 1/16 inch ≈ 1.6 mm) and its inner diameter (ID_{cat}, 0.5 mm) as

$$f = \frac{S_{\mathrm{ref}}}{S_{\mathrm{HP}}} = \frac{{ID_{\mathrm{tube}}}^2}{{ID_{\mathrm{tube}}}^2 - \left({OD_{\mathrm{cat}}}^2 - {ID_{\mathrm{cat}}}^2\right)} = \frac{4^2}{4^2 - (1.6^2 - 0.5^2)} \approx 1.17.$$

When series of ^{15}N NMR spectra were acquired using a series of low flip angle θ_{RF} RF pulses (e.g., for measurement of T_1 or $T_{1\rho}$), the following formula was used to adjust for polarization depletion due to regular pulsing:

$$SE_{15\mathrm{N}} = \frac{I_{\mathrm{HP}}}{I_{\mathrm{ref}}} \cdot \frac{c_{\mathrm{ref}}}{c_{\mathrm{MNZ},i}} \cdot \frac{h}{h_0} \cdot \frac{\cos^{n-1}(\theta_{RF})}{\sin(\theta_{RF})} \cdot f$$

where n is the number of the RF pulse applied in the series.

The ^{15}N NMR spectra acquired at 7.05 T (300 MHz MRI scanner) were processed differently. Integral intensities I_{HP} of the NMR signals of ^{15}N nuclei of [$^{15}N_3$]MNZ were at first recalculated to ^{15}N NMR signal enhancement factors $SE_{15\mathrm{N}}$ as

$$SE_{15\mathrm{N}} = \frac{I_{\mathrm{HP}}}{I_{\mathrm{ref}}} \cdot \frac{n_{\mathrm{ref}}}{n_{\mathrm{MNZ}}} \cdot \frac{1}{\sin(\theta_{RF})}$$

where I_{ref} is the integral of ^{15}N NMR signal of the reference sample (neat [^{15}N]pyridine) thermally equilibrated at the 9.4T detection field, $n_{\mathrm{MNZ}} = c_{\mathrm{MNZ},0}V_0$ is the amount of [$^{15}N_3$]MNZ in the solution ($V_0 = 0.6\ \mathrm{mL}$ and $c_{\mathrm{MNZ},0} = 30\ \mathrm{mM}$), $n_{\mathrm{ref}} = c_{\mathrm{ref}}V_{\mathrm{ref}}$ (c_{ref} is the concentration of the reference nuclei (12.4 M)), and θ_{RF} is the flip angle of the RF pulse. This difference is due to the fact that the whole solution was in the sensitive zone of the ^{15}N MRI coil, and hence proper recalculation had to take into account the amount of ^{15}N nuclei rather than their concentration.

Analysis of SABRE complexes: As [$^{15}N_3$]MNZ is partially bound to the SABRE complexes, to assess correct concentration of different species of [$^{15}N_3$]MNZ present in the solution we have to know concentration of the SABRE complexes (an example of ^{1}H NMR spectrum from which they may be determined is provided in Figure S2 in the following section). While different SABRE complexes are present in the solution, the majority is represented by complexes *fac*-[IrH_2IMes([$^{15}N_3$]MNZ)$_3$] (**I**), [IrH_2IMes([$^{15}N_3$]MNZ)$_2$Cl] (**II**), and [IrH_2IMes([$^{15}N_3$]MNZ)$_2$(CD_3OD)] (**III**). Assuming that other hydride complexes also have 2 hydride ligands each, we then attribute total integral intensity of hydride ^{1}H NMR signals $I_{\mathrm{H,total}}$ to c_{total} of 4 mM of hydride atoms. The concentration of SABRE complexes **1**–**3** ($c_{\mathbf{1-3}}$) is then determined as

$$c_{\mathbf{1}} = c_{\mathrm{total}} \cdot \frac{I_{\mathrm{H},\mathbf{1}}}{2I_{\mathrm{H,total}}};\ c_{\mathbf{2}} = c_{\mathrm{total}} \cdot \frac{I_{\mathrm{H},\mathbf{2}} + I'_{\mathrm{H},\mathbf{2}}}{2I_{\mathrm{H,total}}};\ c_{\mathbf{3}} = c_{\mathrm{total}} \cdot \frac{I_{\mathrm{H},\mathbf{3}} + I'_{\mathrm{H},\mathbf{3}}}{2I_{\mathrm{H,total}}}$$

where $I_{\mathrm{H},\boldsymbol{i}}$ is the integral intensity of ^{1}H NMR signal of corresponding (as complexes **2** and **3** have magnetically inequivalent hydrides, this is represented by additional integral intensities $I'_{\mathrm{H},\mathbf{2}}$ and $I'_{\mathrm{H},\mathbf{3}}$). From this, concentrations $c_{\mathrm{MNZ},i}$ of different forms of [$^{15}N_3$]MNZ are given by

$$c_{\mathrm{MNZ,ax}} = 2\ \mathrm{mM};\ c_{\mathrm{MNZ,eq}} = c_{\mathbf{1}} + c_{\mathrm{MNZ,ax}};\ c_{\mathrm{MNZ,nb}} = c_{\mathrm{MNZ,total}} - c_{\mathrm{MNZ,eq}} - c_{\mathrm{MNZ,ax}}$$

where $c_{\mathrm{MNZ,ax}}$ is the concentration of axially bound [$^{15}N_3$]MNZ, $c_{\mathrm{MNZ,eq}}$ is the concentration of equatorially bound [$^{15}N_3$]MNZ, and $c_{\mathrm{MNZ,nb}}$ is the concentration of non-bound ("free") [$^{15}N_3$]MNZ.

Among ^{15}N NMR signals of bound [$^{15}N_3$]MNZ, signals of ^{15}N nuclei in N1 position of the imidazole cycle strongly overlap with those of non-bound [$^{15}N_3$]MNZ (unlike those of ^{15}N nuclei in N3 and $\underline{N}O_2$ positions). To estimate their contribution to the integral intensity, similarly to previously published work,[2] we assumed that $I_{15\mathrm{N1,eq}} \approx I_{15\mathrm{N3,eq}} \approx I_{15\mathrm{NO2,eq}}$ and $I_{15\mathrm{N1,ax}} \approx I_{15\mathrm{N3,ax}} \approx I_{15\mathrm{NO2,ax}}$. Additionally, $I_{15\mathrm{N1,b}} = \frac{I_{15\mathrm{N3,b}} + I_{15\mathrm{NO2,b}}}{2}$. Thus, contribution of non-bound ^{15}N1 nuclei $I_{15\mathrm{N1,nb}}$ to total integral intensity $I_{15\mathrm{N1,total}}$ was determined as

$$I_{15\mathrm{N1,nb}} = I_{15\mathrm{N1,total}} - I_{15\mathrm{N1,b}} = I_{15\mathrm{N1,total}} - \frac{I_{15\mathrm{N3,b}} + I_{15\mathrm{NO2,b}}}{2}.$$

^{15}N hyperpolarization values: Finally, ^{15}N polarization values $P_{15\mathrm{N}}$ were calculated as

$$P_{15\mathrm{N}} = SE_{15\mathrm{N}} \cdot P_{15\mathrm{N,therm}} = SE_{15\mathrm{N}} \cdot \frac{\gamma_{15\mathrm{N}}\hbar B_0}{2k_BT}$$

where $P_{15\mathrm{N,therm}}$ is the thermal polarization of ^{15}N nuclei ($3.22\cdot10^{-4}$ % in in 9.4 T or $2.4\cdot10^{-4}$ % in in 7.05 T), $\gamma_{15\mathrm{N}}$ is the gyromagnetic ratio of ^{15}N nuclei, $\hbar$ is the Planck constant, B_0 is the magnetic field (9.4 T or 7.05 T), k_B is the Boltzmann constant, and T is the temperature of the sample.

Hydride complexes in activated SABRE solution of [$^{15}N_3$]MNZ

To determine the SABRE complexes responsible for polarization transfer to [$^{15}N_3$]MNZ and estimate their concentrations, ^{1}H NMR spectra of the hydride complexes present in several samples used for the SABRE experiments on different days were acquired. Firstly, high-field SABRE experiments were conducted; the NMR signals of the hydrides were acquired by applying a 45° RF pulse immediately after bubbling *p*-H_2 for 35 s through the solution positioned in the 9.4 T NMR system (Figure S2a–c). The ^{1}H NMR spectrum of thermally polarized hydride complexes was acquired overnight under ~7 bar pressure of H_2 with catheter inserted in the NMR tube (Figure S2d). The pressurization with H_2 was deemed necessary as without it HD-exchange took place and even after overnight acquisition the ^{1}H NMR signals were too weak for proper analysis due to presence of additional $^{2}J_{H,D}$-couplings; additionally, without H_2 atmosphere, degradation of hydride complexes due to O_2 and H_2O presence happened.

It is important to note that in the high-field SABRE experiments the shapes of the resultant ^{1}H NMR signals are dictated by the *J*-coupling constants between the *p*-H_2-nascent protons and other magnetic nuclei in the relevant SABRE complexes. Here, we observed signals additional to ones previously reported by Yi et al. for the SABRE solution of MNZ in CD_3OD.[2] This may be explained by (*i*) usage of MNZ at higher concentration (30 mM in our work vs. 20 mM in the previous work) and (*ii*) presence of fully ^{15}N-labelled MNZ in the SABRE complexes. While reason (*i*) should mostly affect the ratio of complexes **I**–**III**, it apparently also leads to formation of other hydride complexes, as also evident from Figure 2d. In a recent SABRE study of ornidazole antibiotic (analogue of metronidazole) the ratio of SABRE-active complexes was similarly changed when amount of ornidazole relative to amount of Ir-catalyst present in the solution was increased; unidentified complexes also appeared at higher concentration of ornidazole.[4] The reason (*ii*) leads to splitting of ^{1}H NMR signals due to $^{2}J_{1H,15N}$-couplings as well as “activation” of symmetric hydride complexes like **I** in PASADENA experiments–when MNZ with natural abundance of ^{15}N nuclei is used, the protons in complex **I** are magnetically equivalent and only <1% of **I** contain one ^{15}N nucleus bound to Ir-center, producing AA'B three-spin system. In our case, AA'BB' four-spin systems are present, and magnetically inequivalent (but chemically equivalent) hydrides become active in high-field SABRE spectra.

The assignment of ^{1}H NMR signals was done on the basis of previous work.[2] Similar unassigned ^{1}H NMR signals were found in one of the previous works on SABRE-SHEATH with non-labelled MNZ, however, they appear to be very weak.[5] The determination of nature of these complexes will require future studies likely involving MNZ introduced in the SABRE solution in different ratios to the SABRE catalyst precursor. The fractions of complexes **1**, **2** and **3** were found to be 55, 21, and 9 %, respectively (the remainder of ~15% is attributed to other hydride species present in the thermally polarized sample).

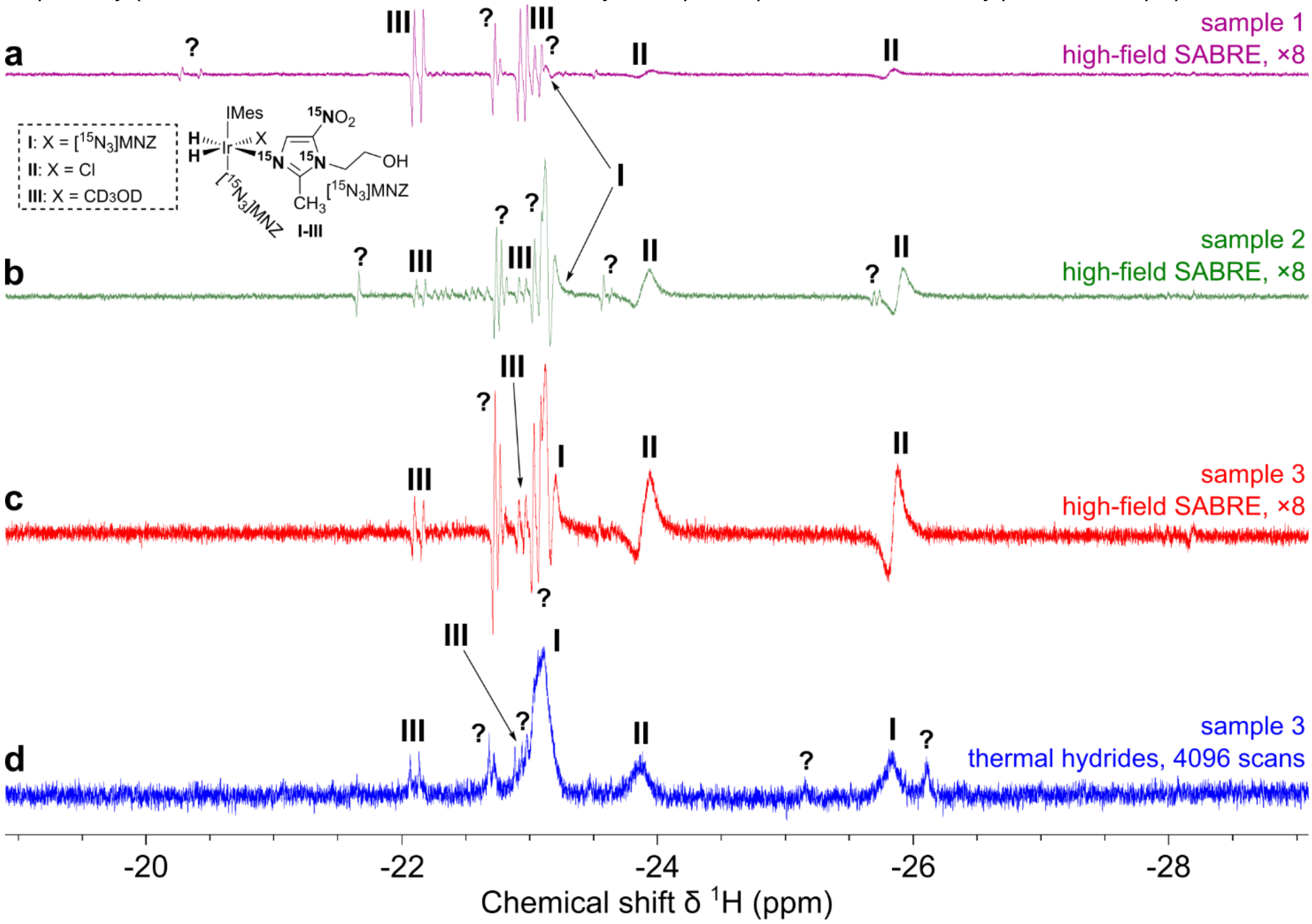

Figure S2. (**a**–**c**) ^{1}H NMR high-field SABRE spectra of activated solutions studied on different days. The spectra were acquired using 45° RF pulse immediately after the end of 35 s-long bubbling of *p*-H_2 through the solution positioned in 9.4 T (inside the NMR spectrometer). These spectra were acquired at the end of the respective experimental days. (**d**) ^{1}H NMR spectrum of activated sample 3 (corresponding to ^{1}H NMR PASADENA spectrum presented in panel (c)).

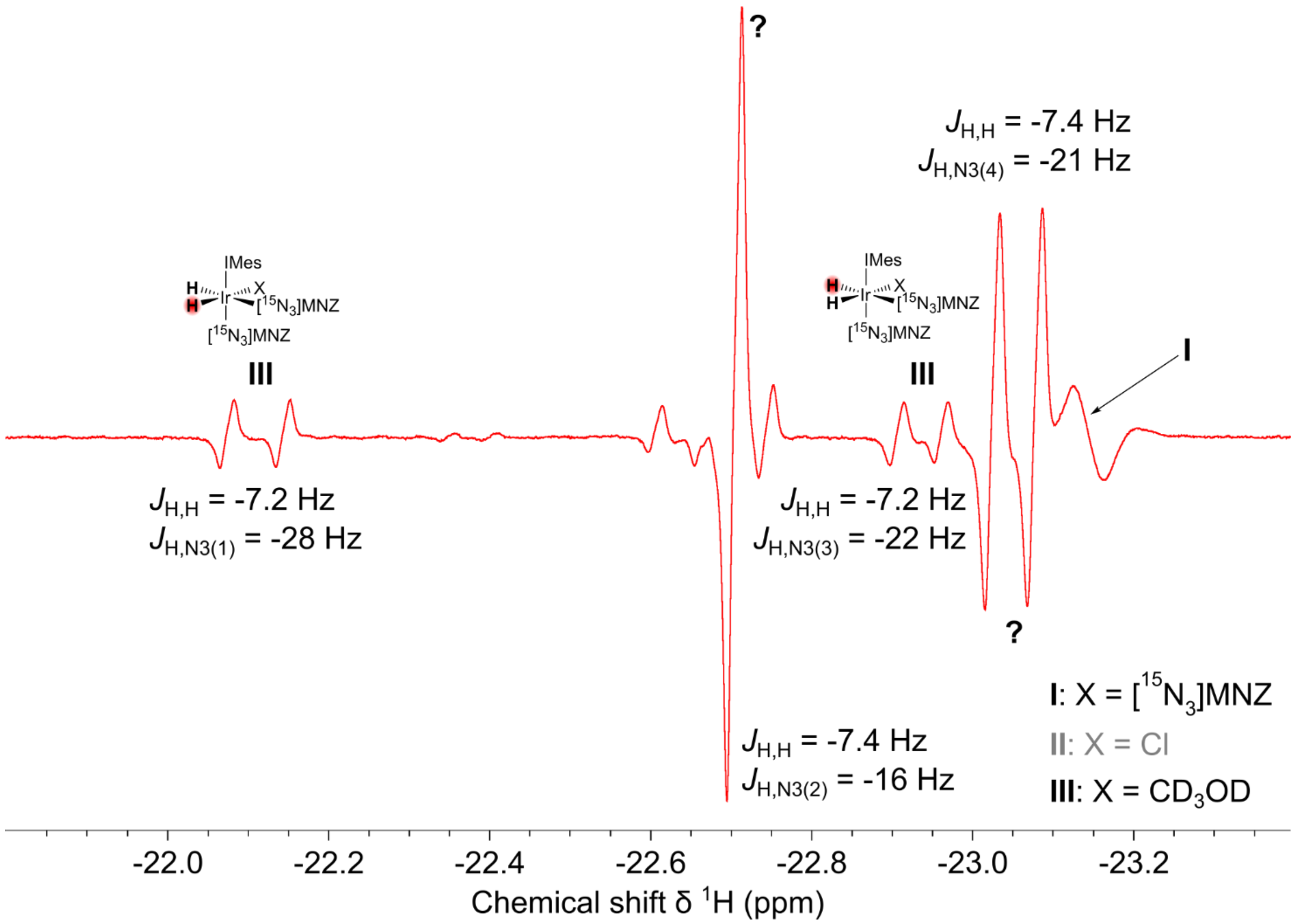

Figure S3. *J*-couplings observed in the ^{1}H NMR spectra of hydrides of polarization transfer complexes **I** and **III** hyperpolarized via PASADENA (signals of **II** are outside of the presented region). Prominent PASADENA signals of an unidentified PTC "**?**" (for SABRE complexes, two hydride signals are typically present) and its distinct *J*-couplings are also evident.

^{15}N polarization levels dependence on magnetic field B_0 in SLIC-SABRE

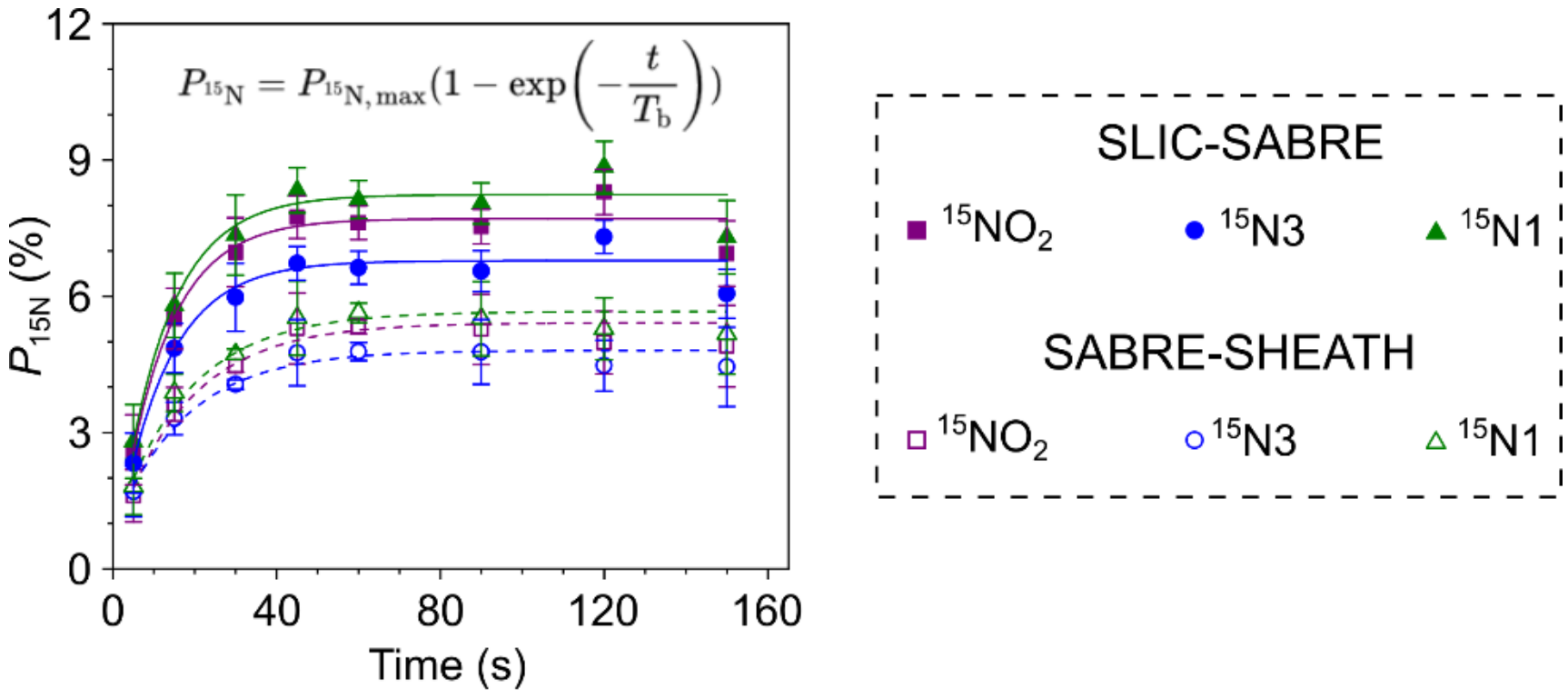

Group	T_b, s		$P_{^{15}N,max}$, %		SLIC improvement factor	R^2	
	SHEATH	SLIC	SHEATH	SLIC		SHEATH	SLIC
$^{15}NO_2$	18±3	12±3	5.4±0.1	7.7±0.2	1.43±0.06	0.966	0.941
$^{15}N3$	17±2	12±4	4.8±0.1	6.8±0.2	1.42±0.07	0.965	0.917
$^{15}N1$	18±3	12±3	5.7±0.2	8.2±0.2	1.44±0.09	0.959	0.939

Figure S4. ^{15}N polarization buildup in SLIC-SABRE (B_0 = 50 µT, B_1 = 12 µT) and SABRE-SHEATH (B_0 = 0.71 µT) conditions, exponential fitting parameters, and SLIC-to SHEATH improvements factor derived from the fitted data. Each experiment was repeated N = 3 times.

^{15}N polarization levels dependence on magnetic field B_0 in SLIC-SABRE

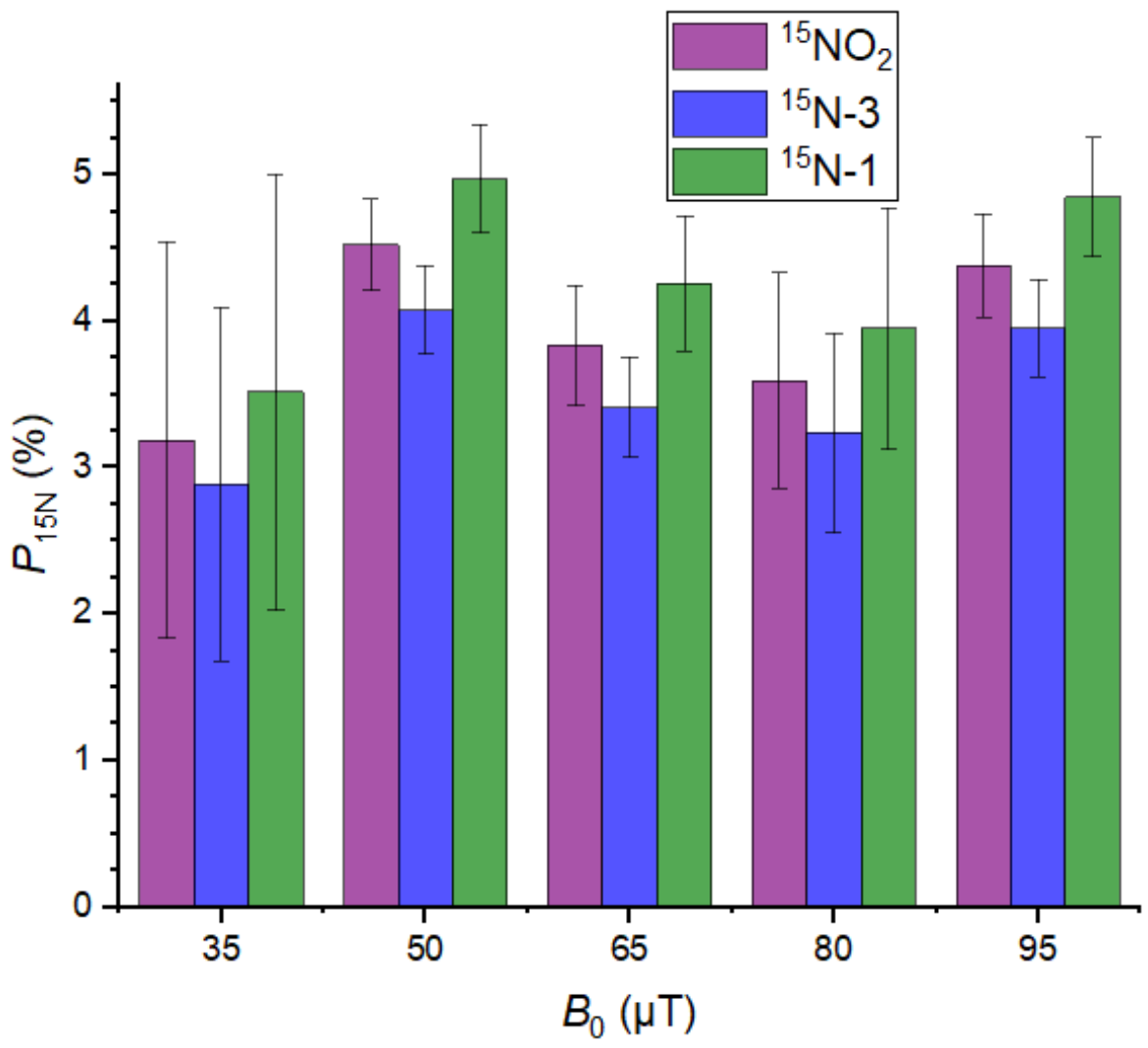

Figure S5. ^{15}N polarization dependence on static shield B_0 in SLIC-SABRE protocol (B_1 of 7.5 µT was applied). No significant dependence exists in the range of investigated B_0 values. 70%-enriched p-H_2 was used for the experiments (see Figure S10).

T_1 relaxation times of ^{15}N nuclei in [$^{15}N_3$]metronidazole in SABRE solution

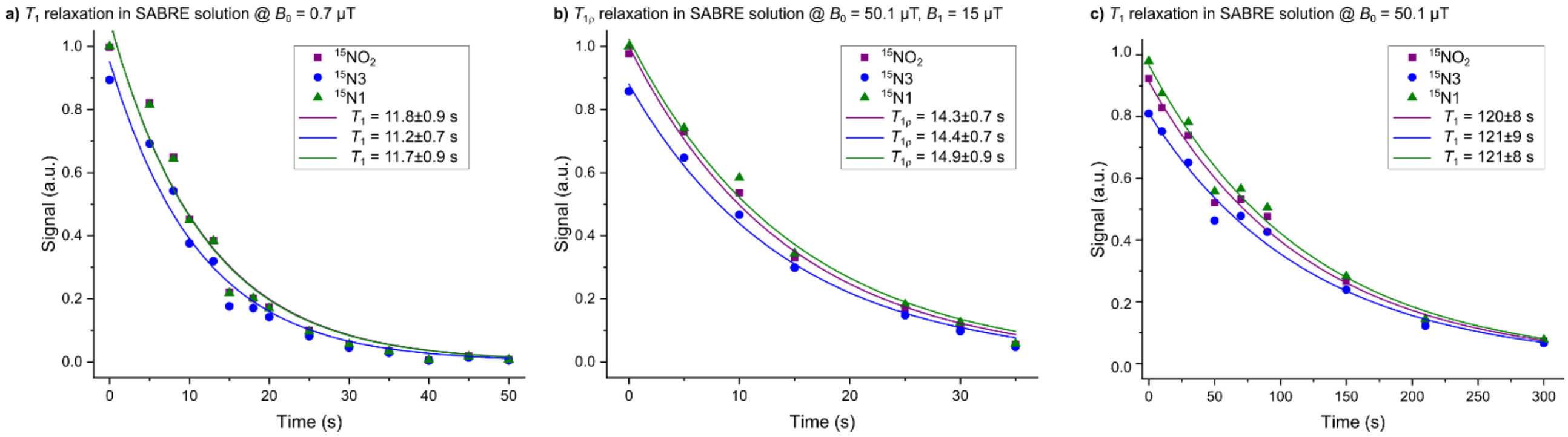

Figure S6. (**a**) Relaxation dynamics of ^{15}N polarization of free [$^{15}N_3$]MNZ in SABRE-SHEATH conditions (B_0 = 0.7 μT, no B_1). (**b**) Relaxation dynamics of ^{15}N polarization of free [$^{15}N_3$]MNZ in SLIC-SABRE conditions (in B_0 = 50 μT, B_1 = 15 μT is applied during relaxation). (**c**) Relaxation dynamics of ^{15}N polarization of free [$^{15}N_3$]MNZ in B_0 = 50 μT in absence of any SLIC pulse.

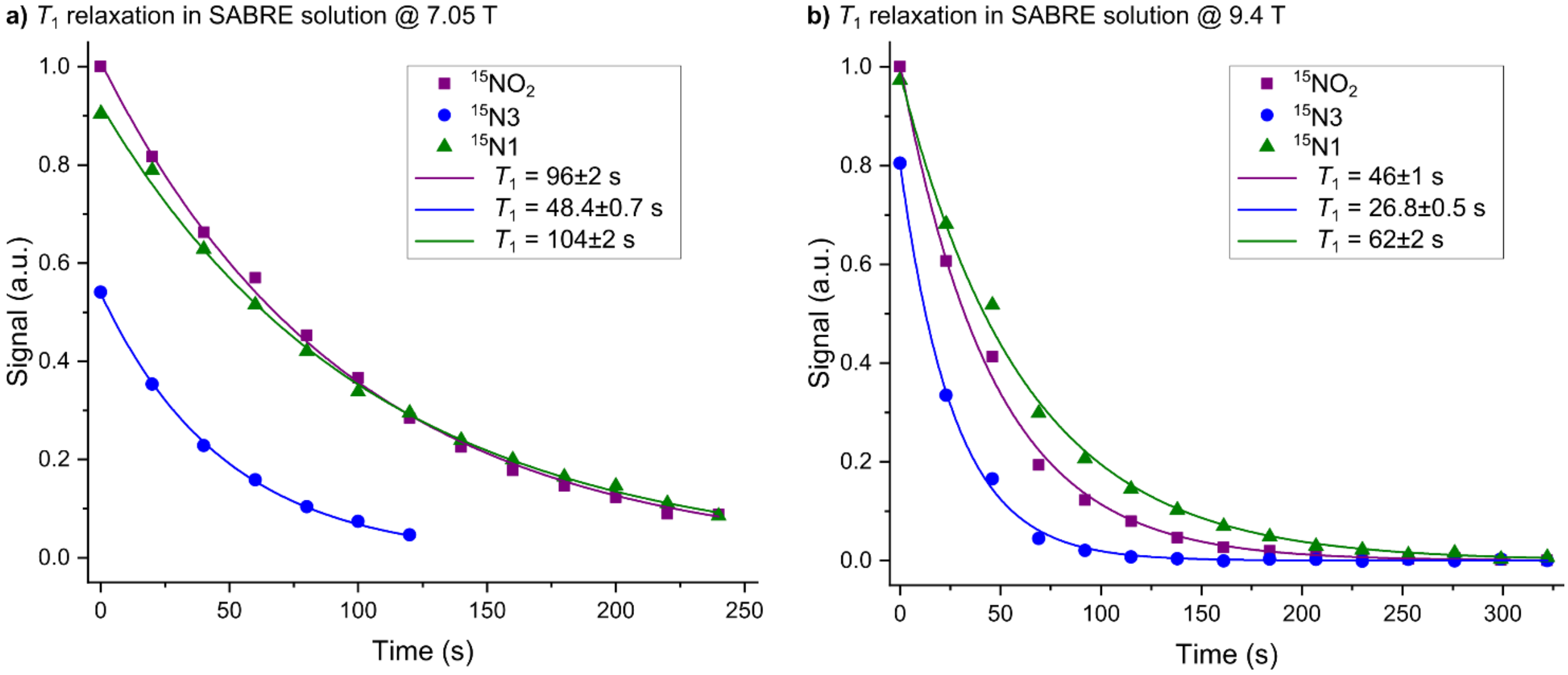

Figure S7. Relaxation dynamics of ^{15}N polarization of free [$^{15}N_3$]MNZ at B_0 = 7.05 and 9.4 T.

Thermal ^{15}N NMRD measurements

Sample compositions

106.9 mM [$^{15}N_3$]MNZ was dissolved as a saturated solution (concentration estimated, see below) in 550 μL methanol-d$_4$ (Sigma-Aldrich, 441384). In the other case, 38.5-39 mM [$^{15}N_3$]MNZ was dissolved as a saturated solution (concentration estimated, see below) in 550 μL D_2O (Deutero, 00506), then 62.1 mM Trizma (Tris) buffer (Sigma-Aldrich, T7943) and 1.4 mM ethylendiamintetracetic acid (EDTA, SERVA, 11280.02) were added, and the final pH was 7.5. In both cases, the sample was degassed inside the NMR tube using Argon gas for 3 minutes, then the tube was sealed using an NMR cap and Parafilm.

Concentrations for saturated MNZ solutions were estimated by adding more MNZ to the solution until a precipitate appeared. Achieved concentration was estimated by comparing to a ^{15}N-spectrum with a known concentration of pyridine (**Tab. S1**), all acquired with 90° flip angle and RG 18.

Table S1. Estimated concentrations for [$^{15}N_3$]MNZ in methanol-d$_4$ and water-d$_2$ using a [^{15}N]pyridine spectrum with a known concentration of 100.9 mM.

Substrate	Integral, a.u.	N scans	Concentration, mM	Comment
Pyridine in H_2O	3499.82	1	100.94	reference
MNZ in MeOD	3707.93	1	106.94	calculated
MNZ in D_2O w/Tris	10668.03	8	38.46	calculated
MNZ in D_2O	10885.66	8	39.24	calculated

SWAP-enhanced NMRD experiment

A ^{15}N SWAP-IR sequence with proton decoupling during FID was used with the following parameters: *d1* = 15 s, *aq* = 2.95 s, *sw* = 397.7 ppm, *o1p* = 259 ppm, *o2p* = 4 ppm. The SWAP-IR sequence[6] was used without changes and without phase cycling:

^{15}N: 90_X–τ–180_X–τ–90_X90_Y–τ–180_X–τ–90_Y–(1 ms, PFG SMSQ10.100, 11%)–vd–90_X–FID

^{1}H: 90_X–τ–180_X–τ–90_X90_Y–τ–180_X–τ–90_Y————————————————————decoupling

The SWAP is almost identical to the INEPT with refocusing, but should provide distortion-free lines since it does not populate multiplet spin order. As a result of this excitation, MNZ signals are polarized.

The following *vd* list was used for methanol-d_4: 0, 0.5, 1, 2, 3, 4, 5, 6, 8,12, 16, 32, 64, 96, 128, 192, 256, 512, 768, 1536 s.

The following *vd* list was used for D_2O: 0, 0.5, 1, 2, 3, 4, 5, 6, 8, 12, 16, 32, 64, 96, 128, 192, 256, 384, 768 s.

The optimum SWAP interpulse delay, τ, was calibrated for each sample experimentally (Figure S1), both leading to optimum τ = 20 ms. In methanol-d_4, the NO_2 T_1 NMRD was observed using thermal acquisition without SWAP enhancement.

Subsequent MFC experiment followed according to the following sequence: (1) ^{1}H thermal relaxation at B_0 for D_{B0} = 19 s, (2) SWAP sequence to transfer ^{1}H polarization to longitudinal polarization of ^{15}N3 and ^{15}N1 of MNZ, (3) shuttling to desired low-field, either inside the NMR bore or in the solenoid electromagnet within the magnetic shield and with appropriately set current, (4) waiting for ^{15}N relaxation at low-field for D_{LF}, (5) shuttling to B_0 followed by settling delay of 150 ms, (6) ^{15}N 90° excitation and signal acquisition with ^{1}H decoupling.

The SWAP calibration curve of the MNZ sample in methanol-d_4 is shown in Figure S1.

All extracted NMRD values are shown in Table S2 and Figure S7. T_1 relaxation rates were fitted with mono-exponential decay curves in Origin Lab Pro (version 2021b).

^{15}N MR signals were acquired using a 9.4 T wide-bore NMR (WB400, Avance NEO, Bruker) with a 5 mm BBFO probe. The temperature of the NMR system was set to 293 K. The room temperature was maintained.

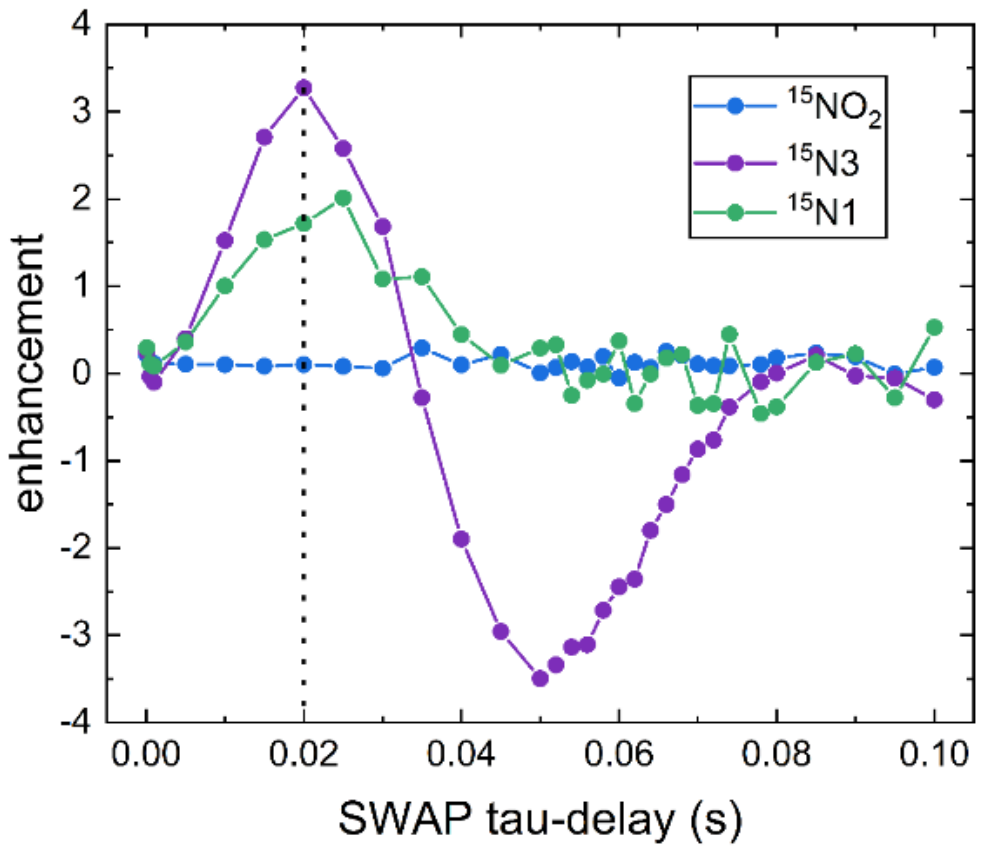

Figure S8. SWAP calibration curve. This experiment was used to find the optimum value for the interpulse delay (2× τ) to transfer the ^{1}H polarization to the ^{15}N of MNZ. A maximum enhancement compared to the thermal ^{15}N spectrum of 3.3 is visible at a τ = 20 ms for N3 and of 2.0 at a τ = 25 ms for N1. No enhancement was visible for NO_2. A τ = 20 ms was chosen. Such a calibration was performed for both samples, leading to similar results.

Table S2. T_1 and standard deviation, s.d., (in seconds) from nuclear magnetic resonance dispersion curves NMRD for [$^{15}N_3$]MNZ samples. A mono-exponential curve was used for T_1-fitting. Note, that in some cases, $^{15}NO_2$ signal was not observed.

**B_0, T**	**[$^{15}N_3$]MNZ in methanol-d_4 (Figure 3c)**					
	$^{15}NO_2$		^{15}N3		^{15}N1	
	T_1	s.d. T_1	T_1	s.d. T_1	T_1	s.d. T_1
$7.8\cdot10^{-6}$	10.4261	2.68312	0.63652	0.15028	4.60619	0.76439
$3.1\cdot10^{-5}$	9.58323	2.58184	1.02083	0.36197	3.23599	0.49102
$1.3\cdot10^{-4}$	10.86313	2.72259	1.21283	0.42802	3.85784	0.56374
$5\cdot10^{-4}$	13.40211	3.66789	1.50317	0.5177	3.16344	0.45058
$2\cdot10^{-3}$	8.01073	1.80147	1.0715	0.32272	4.11257	0.57802
$4\cdot10^{-3}$	21.74023	5.80912	2.05998	0.60563	5.1824	0.77408
0.032	534.4593	201.1249	2.40968	0.18692	26.72335	2.41598
0.064	576.2213	223.5035	5.33109	0.32534	106.9485	9.22082
0.128	264.2196	87.70774	12.23329	0.75448	181.702	17.37569
0.256	386.1559	145.8224	28.29419	2.00876	220.3565	21.0768
0.512	387.7653	114.4896	40.62446	2.79811	191.2497	16.74225
0.768	713.7115	324.1204	46.6731	3.08593	225.4527	18.46849
1.024	1085.193	521.2119	48.3994	3.16271	193.3431	16.13714
1.536	772.4165	301.7078	47.9384	3.0875	186.3518	14.70306
2.048	478.2899	105.6556	48.59398	3.01599	225.3539	17.1298
3.072	359.7498	64.96644	49.48341	2.84496	184.3116	12.20095
4.096	234.309	34.95602	44.39012	2.44163	170.1915	10.23597
6.144	161.9769	18.88951	34.84684	1.73127	152.436	7.64762
8.192	114.3011	10.48965	29.91383	1.3625	114.7702	5.15035
9.4	79.22546	6.70283	27.94726	1.17185	107.5923	4.31559
**B_0, T**	**[$^{15}N_3$]MNZ in Tris-buffered water-d_2 (Figure 3b)**					
	$^{15}NO_2$		^{15}N3		^{15}N1	
	T_1	s.d. T_1	T_1	s.d. T_1	T_1	s.d. T_1
$7.8\cdot10^{-6}$	28.11979	11.33742	8.70903	1.63413	12.61388	2.80946
$3.1\cdot10^{-5}$	41.78447	20.71128	11.53675	2.78912	14.06656	3.72689
$1.3\cdot10^{-4}$	83.67068	42.11989	8.90993	1.90159	23.02107	7.07011

5·10⁻⁴	56.56688	28.57704	12.16766	3.01417	22.85624	6.34442
2·10⁻³	86.58096	47.43979	13.30096	2.81252	25.76888	7.84507
4·10⁻³	31.29635	17.92611	11.29376	2.43237	22.00355	6.03918
0.032	no signal	no signal	27.43726	5.7927	60.63268	15.38671
0.064	no signal	no signal	31.33701	6.46562	120.2024	34.1101
0.128	no signal	no signal	55.327	9.80056	103.5098	28.01593
0.256	no signal	no signal	92.4732	16.33693	211.1213	54.85202
0.512	no signal	no signal	106.259	20.62225	132.2465	34.98923
0.768	no signal	no signal	124.9765	25.15961	114.5136	31.6933
1.024	no signal	no signal	115.5924	20.94306	127.0953	28.75692
1.536	no signal	no signal	69.04141	12.5587	174.5734	46.68576
2.048	no signal	no signal	84.45577	16.42635	183.7971	47.57424
3.072	no signal	no signal	78.21302	13.46436	165.8903	34.6447
4.096	226.0098	190.0271	225.9741	196.5842	87.41831	16.81151
6.144	409.1714	264.825	409.1714	274.0151	97.40276	17.64441
8.192	66.26664	24.83992	66.26663	25.70192	82.31809	13.57144
9.4	97.59414	27.54045	97.59414	28.49617	88.03248	12.38422
**B_0, T**	**[$^{15}N_3$]MNZ in water-d_2 w/o buffer (Figure 3a)**					
	$^{15}NO_2$		$^{15}N3$		$^{15}N1$	
	T_1	s.d. T_1	T_1	s.d. T_1	T_1	s.d. T_1
7.8·10⁻⁶	9.28487	3.55696	1.07034	0.4156	2.79467	0.90952
3.1·10⁻⁵	9.4119	3.51698	3.275	1.15319	5.27912	1.72155
1.3·10⁻⁴	15.0254	7.36671	1.95819	1.14587	4.88843	1.45747
5·10⁻⁴	13.77255	7.58888	2.34698	1.0671	6.44888	2.22668
2·10⁻³	22.59441	15.74535	3.9384	1.5935	4.94596	1.64002
4·10⁻³	49.94764	46.76538	2.86609	1.10231	8.2692	3.30977
0.032	no signal	no signal	4.20085	0.8372	48.23863	13.98009
0.064	no signal	no signal	11.00391	2.11094	106.9308	32.42246
0.128	no signal	no signal	25.21938	5.88702	129.7237	43.06317
0.256	no signal	no signal	68.63667	15.43442	229.704	96.09677
0.512	no signal	no signal	60.48624	12.69168	138.457	38.21293
0.768	no signal	no signal	62.43187	15.98993	198.2961	69.51885
1.024	no signal	no signal	88.17838	19.67843	212.6903	77.13649
1.536	no signal	no signal	63.07826	13.03633	253.7972	87.52594
2.048	no signal	no signal	51.1503	11.99426	155.2118	44.84661
3.072	no signal	no signal	105.0213	19.12729	155.8905	39.81465
4.096	70.78282	76.29779	64.21253	11.84139	97.30905	26.11785
6.144	88.07409	46.63027	35.88085	6.18738	113.3794	26.6361
8.192	140.841	50.82641	33.8153	5.29738	83.94137	15.31179
9.4	56.47224	15.39848	21.25158	3.22296	102.5335	20.39905

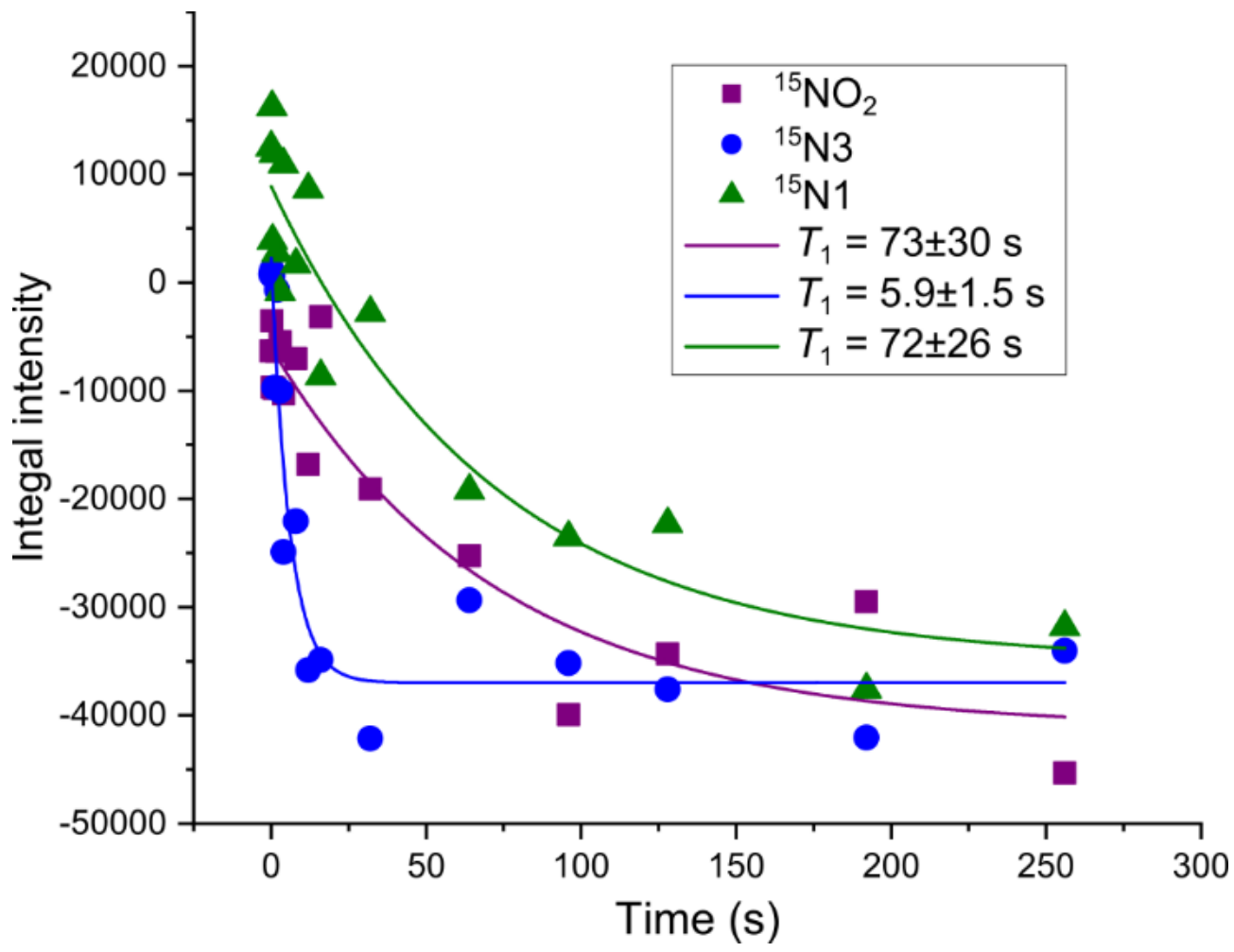

Figure S9. ^{15}N T_1 relaxation of 38 mM [$^{15}N_3$]MNZ in CD_3OD in B_0 = 9.4 T.

Quantification of methanol content after purification

To determine fraction of residual methanol in the samples after evaporation, the samples were analyzed via ^2H NMR spectroscopy and the integral intensities of labile deuterons $I(\mathbf{D}_2O)$ and trideuteromethyl droup $I(C\mathbf{D}_3OD)$ were used to calculate molar fraction of methanol $\chi(CD_3OD)$ and to estimate its concentration $c(CD_3OD)$ after evaporation:

$$\chi(CD_3OD) = f\frac{{}^1\!/_3\, I(C\mathbf{D}_3OD)}{{}^1\!/_2\left(I(\mathbf{D}_2O) - {}^1\!/_3\, I(C\mathbf{D}_3OD)\right)};\, c(CD_3OD) \approx \chi(CD_3OD)\cdot c(D_2O) = 55.56\chi(CD_3OD)\text{ M.}$$

where f is a dilution factor (1.5–3) as some samples were diluted with D_2O for the measurements by a known factor to properly fill the sensitive zone of the NMR probe. Admittedly, at high concentrations (>1 M) this formula stops being accurate and is used to represent the order of magnitude at which the residual methanol is present in the solution

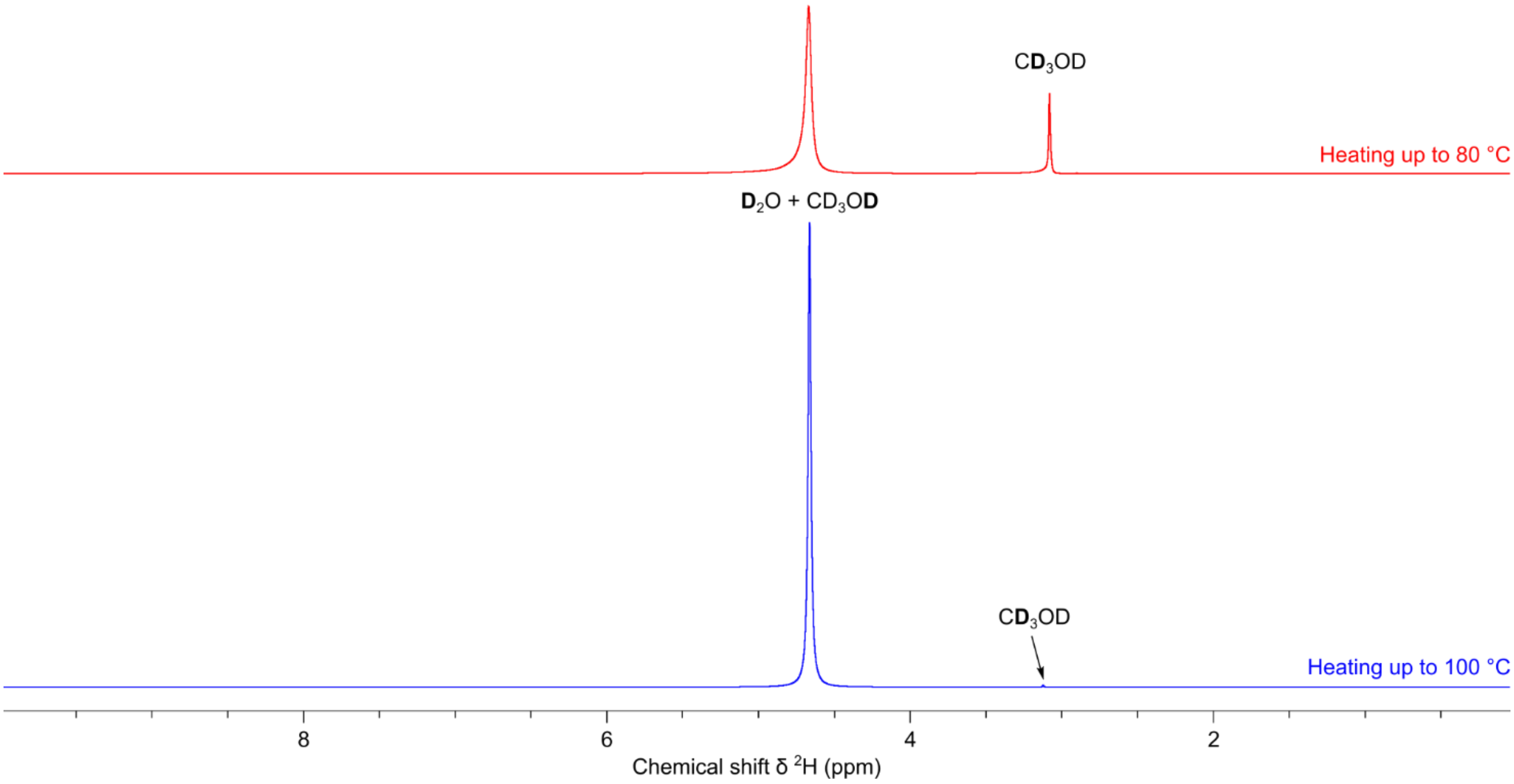

Figure S10. Examples of ^{2}H NMR spectra of the samples after methanol evaporation for 15 s at 80 °C (red) and 100 °C (blue).

Table S3. Molar fractions of methanol-d$_4$ after evaporation in different experiments and the ^{2}H NMR signal integral intensity values from which they were calculated.

Temperature, °C	$I(\mathbf{D}_2\mathrm{O})$	$I(\mathrm{C}\mathbf{D}_3\mathrm{OD})$	$\chi(\mathrm{CD_3OD})$, %	$c(\mathrm{CD_3OD})$, M	Dilution factor f
80	1	0.0746	5.10	2.83	1
80	1	0.1801*	12.8	7.10	1
80	1	0.0878	6.03	3.35	1
80	1	0.0879	6.04	3.35	1
100	1	0.0009	0.06	$33.3 \cdot 10^{-3}$	1
100	1	0.0004	0.0533	$29.6 \cdot 10^{-3}$	2
100	1	0.0004	0.08	$44.5 \cdot 10^{-3}$	3
100	1	0.0007	0.0934	$51.9 \cdot 10^{-3}$	2
100	1	0.0003	0.04	$22.2 \cdot 10^{-3}$	2
100	1	0.0002	0.02	$11.1 \cdot 10^{-3}$	1.5
100	1	0.0005	0.05	$27.8 \cdot 10^{-3}$	1.5
**Average**					
80	×	×	6.0±0.4	3.2±0.2	×
100	×	×	0.06±0.02	$(32 \pm 12) \cdot 10^{-3}$	×

* A falling out data point (was not used for calculation of the average).

Parahydrogen enrichment quantification

To determine fraction of p-H_2 in the produced batches of *para*-enriched hydrogen the following procedure was used. Consecutively, ^1H free induction decay (FID) signals of *para*-enriched hydrogen, nitrogen (for baseline) and thermally polarized hydrogen were obtained (256 scans, each gas at 5 bar pressure). Then magnitude adjusted ^1H NMR spectra of *para*-enriched and thermally polarized hydrogen were produced by subtracting the N_2 baseline from the initial spectra. Then the p-H_2 fraction is determined as

$$\chi(pH_2) = \left(1 - \frac{3}{4} \cdot \frac{I(pH_2)}{I(oH_2)}\right) \cdot 100\%$$

where $I(pH_2)$ and $I(oH_2)$ are integral intensities of ^1H NMR signals *para*-enriched and thermally polarized hydrogen.

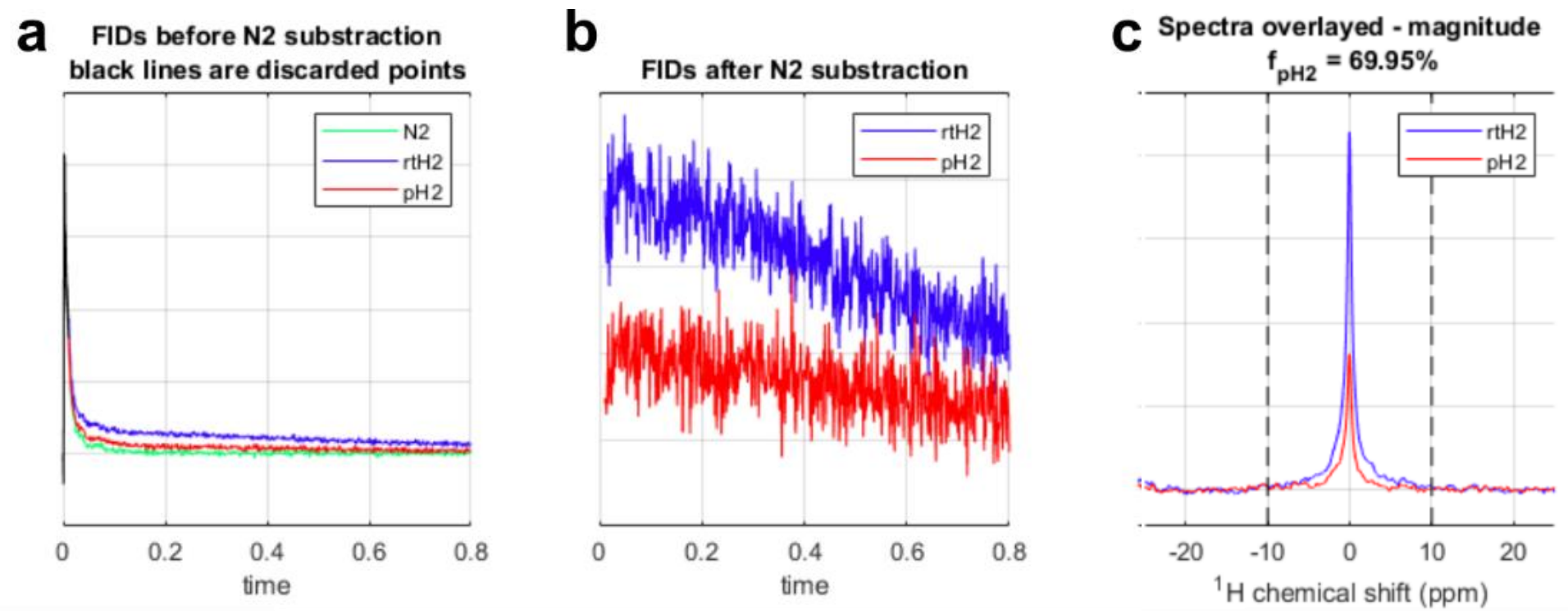

Figure S11. An example of p-H_2 fraction quantification procedure for 70% *para*-enriched hydrogen. (**a**) Initial FID signals of nitrogen, *para*-enriched and thermally polarized hydrogen. Black data points are omitted from further calculations. (**b**) Adjusted FID signals of *para*-enriched hydrogen and thermally polarized hydrogen adjusted by subtraction of baseline FID of nitrogen from the initial FID. (**c**) Magnitude ^1H NMR spectra of *para*-enriched hydrogen and thermally polarized hydrogen.

References

(1) Shchepin, R. V.; Birchall, J. R.; Chukanov, N. V.; Kovtunov, K. V.; Koptyug, I. V.; Theis, T.; Warren, W. S.; Gelovani, J. G.; Goodson, B. M.; Shokouhi, S.; Rosen, M. S.; Yen, Y.; Pham, W.; Chekmenev, E. Y. Hyperpolarizing Concentrated Metronidazole $^{15}NO_2$ Group over Six Chemical Bonds with More than 15 % Polarization and a 20 Minute Lifetime. *Chem. – Eur. J.* **2019**, *25* (37), 8829–8836. https://doi.org/10.1002/chem.201901192.

(2) Yi, A. P.; Salnikov, O. G.; Burueva, D. B.; Chukanov, N. V.; Chekmenev, E. Y.; Koptyug, I. V. Solvent Effects in Hyperpolarization of ^{15}N Nuclei in [$^{15}N_3$]Metronidazole and [$^{15}N_3$]Nimorazole Antibiotics via SABRE-SHEATH**. *Anal. Sens.* **2024**, e202400045. https://doi.org/10.1002/anse.202400045.

(3) Sviyazov, S. V.; Burueva, D. B.; Chukanov, N. V.; Razumov, I. A.; Chekmenev, E. Y.; Salnikov, O. G.; Koptyug, I. V. ^{15}N Hyperpolarization of Metronidazole Antibiotic in Aqueous Media Using Phase-Separated Signal Amplification by Reversible Exchange with Parahydrogen. *J. Phys. Chem. Lett.* **2024**, *15* (20), 5382–5389. https://doi.org/10.1021/acs.jpclett.4c00875.

(4) Yi, A. P.; Chukanov, N. V.; Kabir, M. S. H.; Trepakova, A. I.; Chekmenev, E. Y.; Salnikov, O. G.; Koptyug, I. V. ^{15}N NMR Signal Amplification by Reversible Exchange of [$^{15}N_3$]Ornidazole Antibiotic. *J. Phys. Chem. B* **2025**, *129* (44), 11548–11556. https://doi.org/10.1021/acs.jpcb.5c05057.

(5) Kiryutin, A. S.; Yurkovskaya, A. V.; Ivanov, K. L. ^{15}N SABRE Hyperpolarization of Metronidazole at Natural Isotope Abundance. *ChemPhysChem* **2021**, *22* (14), 1470–1477. https://doi.org/10.1002/cphc.202100315.

(6) Linden, N.; Barjat, H.; Kupče, Ē.; Freeman, R. How to Exchange Information between Two Coupled Nuclear Spins: The Universal SWAP Operation. *Chem. Phys. Lett.* **1999**, *307* (3–4), 198–204. https://doi.org/10.1016/S0009-2614(99)00516-3.
